# Dynactin binding to tyrosinated microtubules promotes centrosome centration in *C*. *elegans* by enhancing dynein-mediated organelle transport

**DOI:** 10.1371/journal.pgen.1006941

**Published:** 2017-07-31

**Authors:** Daniel J. Barbosa, Joana Duro, Bram Prevo, Dhanya K. Cheerambathur, Ana X. Carvalho, Reto Gassmann

**Affiliations:** 1 Instituto de Biologia Molecular e Celular, Universidade do Porto, Porto, Portugal; 2 Instituto de Investigação e Inovação em Saúde (I3S), Universidade do Porto, Porto, Portugal; 3 Ludwig Institute for Cancer Research/Dept of Cellular & Molecular Medicine UCSD, La Jolla, CA, United States of America; University of Cambridge, UNITED KINGDOM

## Abstract

The microtubule-based motor dynein generates pulling forces for centrosome centration and mitotic spindle positioning in animal cells. How the essential dynein activator dynactin regulates these functions of the motor is incompletely understood. Here, we dissect the role of dynactin's microtubule binding activity, located in the p150 CAP-Gly domain and an adjacent basic patch, in the *C*. *elegans* zygote. Analysis of p150 mutants engineered by genome editing suggests that microtubule tip tracking of dynein-dynactin is dispensable for targeting the motor to the cell cortex and for generating robust cortical pulling forces. Instead, mutations in p150's CAP-Gly domain inhibit cytoplasmic pulling forces responsible for centration of centrosomes and attached pronuclei. The centration defects are mimicked by mutations of α-tubulin's C-terminal tyrosine, and both p150 CAP-Gly and tubulin tyrosine mutants decrease the frequency of early endosome transport from the cell periphery towards centrosomes during centration. Our results suggest that p150 GAP-Gly domain binding to tyrosinated microtubules promotes initiation of dynein-mediated organelle transport in the dividing one-cell embryo, and that this function of p150 is critical for generating cytoplasmic pulling forces for centrosome centration.

## Author summary

Animal cells rely on molecular motor proteins to distribute intracellular components and organize their cytoplasmic content. The motor cytoplasmic dynein 1 (dynein) uses microtubule filaments as tracks to transport cargo from the cell periphery to the cell center, where the microtubule minus ends are embedded at the centrosome. Conversely, when dynein is anchored at the cell cortex or on organelles in the cytoplasm, the motor can pull on microtubules to position centrosomes within the cell. The intracellular location of centrosomes determines cell geometry and cell fate, and studying the underlying mechanisms will help us understand polarized cell behaviors such as cell migration or neurite outgrowth, and how cleavage plane orientation is established during cell division. Here, we show in *C*. *elegans* embryos that dynactin, an essential dynein regulator, uses its microtubule binding activity to help dynein pull on microtubules for centrosome positioning during the first mitotic division. Our results with engineered dynactin and tubulin mutants suggest that microtubule binding by dynactin increases the efficiency with which dynein can initiate the transport of small organelles towards centrosomes. More organelles moving along microtubules through the viscous cytoplasm means that correspondingly larger pulling forces act on centrosomes. Thus, our work provides evidence for a novel functional link between dynactin's role in initiating transport of dynein cargo and the generation of cytoplasmic pulling forces critical for the positioning of centrosomes.

## Introduction

Cytoplasmic dynein 1 (dynein) is the major microtubule (MT) minus-end directed motor in animals and transports various cargo from the cell periphery to the cell interior. The motor also moves and positions intracellular structures such as nuclei and centrosomes by pulling on the MTs to which they are connected. To generate pulling force, dynein is either attached to anchor proteins fixed at the cell cortex (cortical pulling) [1–4], or dynein is anchored on organelles in the cytoplasm (cytoplasmic pulling) [5,6]. In the latter instance, dynein generates MT length-dependent pulling forces by working against viscous drag as it transports organelles along MTs toward centrosomes.

Dynactin is an essential multi-subunit activator of dynein that forms a tripartite complex with the motor and cargo-specific adaptors proteins [7–11], but how dynactin supports the diverse functions of dynein remains incompletely understood. Dynactin is built around a short actin-like Arp1 filament and has its own MT binding activity, which resides at the end of a long projection formed by the largest subunit p150 [12]. p150 has a tandem arrangement of MT binding regions consisting of an N-terminal cytoskeleton-associated protein glycine-rich (CAP-Gly) domain and an adjacent patch rich in basic residues [13,14]. The CAP-Gly domain binds to MTs and to the MT plus-end tracking proteins (+TIPs) CLIP-170 and end-binding (EB) protein. In animal cells, +TIP binding of dynactin recruits dynein to growing MT ends [9,15–18].

The p150 CAP-Gly domain recognizes the C-terminal EEY/F motif present in α-tubulin and EB/CLIP-170 [19–22]. The C-terminal tyrosine of α-tubulin can be removed and re-ligated in a tyrosination-detyrosination cycle and is proposed to regulate the interactions with molecular motors and other MT binding proteins [23,24]. Tubulin tyrosination is required in mouse fibroblasts to localize CAP-Gly proteins, including p150, to MT plus ends [25], and recent work *in vitro* demonstrated that the interaction between p150's CAP-Gly domain and tyrosinated MTs enhances the initiation of processive dynein motility [26].

The functional significance of MT binding by p150 in animals is best understood in neurons. Single point mutations in the CAP-Gly domain cause the ALS-like motor neuron degenerative disease HMN7B and a form of parkinsonism known as Perry syndrome [27–29]. Cellular and *in vivo* studies addressing the underlying molecular defects revealed that p150 CAP-Gly domain-dependent binding of dynactin to dynamic MTs in the distal axon enhances the recruitment of dynein, which in turn facilitates efficient initiation of retrograde transport [30–32].

While the critical role of p150's CAP-Gly domain in neuronal trafficking is firmly established, little is known about how MT binding by dynactin regulates dynein functions in other cellular contexts. A study in *D. melanogaster* S2 cells reported multipolar spindles with a p150^Glued^ construct lacking the CAP-Gly domain, suggesting a role in organizing MT arrays [33]. In budding yeast, introduction of the motor neuron disease mutation into p150^Nip100^ inhibited the initial movement of the spindle and nucleus into the bud neck during mitosis [34], suggesting that dynactin binding to MTs helps dynein generate pulling forces under load. In budding and fission yeast, dynein is off-loaded to cortical anchors via MTs for subsequent force production, and in budding yeast this requires MT tip tracking of dynein [35–39]. Whether MT tip tracking of dynein plays a role in delivering the motor to the cortex in animal cells remains to be determined.

MT binding of dynactin is significantly enhanced by electrostatic interactions between the p150 basic patch and the acidic tails of tubulins [14,40,41]. In the filamentous fungus *A*. *nidulans*, deletion of the basic patch in p150^NUDM^ diminishes the accumulation of dynactin and dynein at MT tips and partially impairs nuclear migration and early endosome distribution [42]. Interestingly, humans express tissue-specific splice isoforms of p150 that lack the basic patch [43,44], but the implications for dynactin function are unclear.

In the *C*. *elegans* one-cell embryo, dynein and dynactin are essential for centrosome separation, migration of the maternal and paternal pronucleus, centration and rotation of the two pronuclei and the associated centrosomes (the nucleus-centrosome complex, NCC), assembly and asymmetric positioning of the mitotic spindle, chromosome congression, and transversal spindle oscillations in anaphase [45–49]. Here, we use a set of *p150*^*dnc-1*^ and α-tubulin mutants constructed by genome editing to define the role of dynactin's MT binding activity in this system. Our results uncover a functional link between the efficient initiation of dynein-mediated organelle transport, which requires dynactin binding to tyrosinated MTs, and the cytoplasmic pulling forces responsible for centration of centrosomes.

## Results

### Identification of +TIPs required for microtubule plus-end targeting of dynein-dynactin in the *C*. *elegans* early embryo

**Fig 1:**
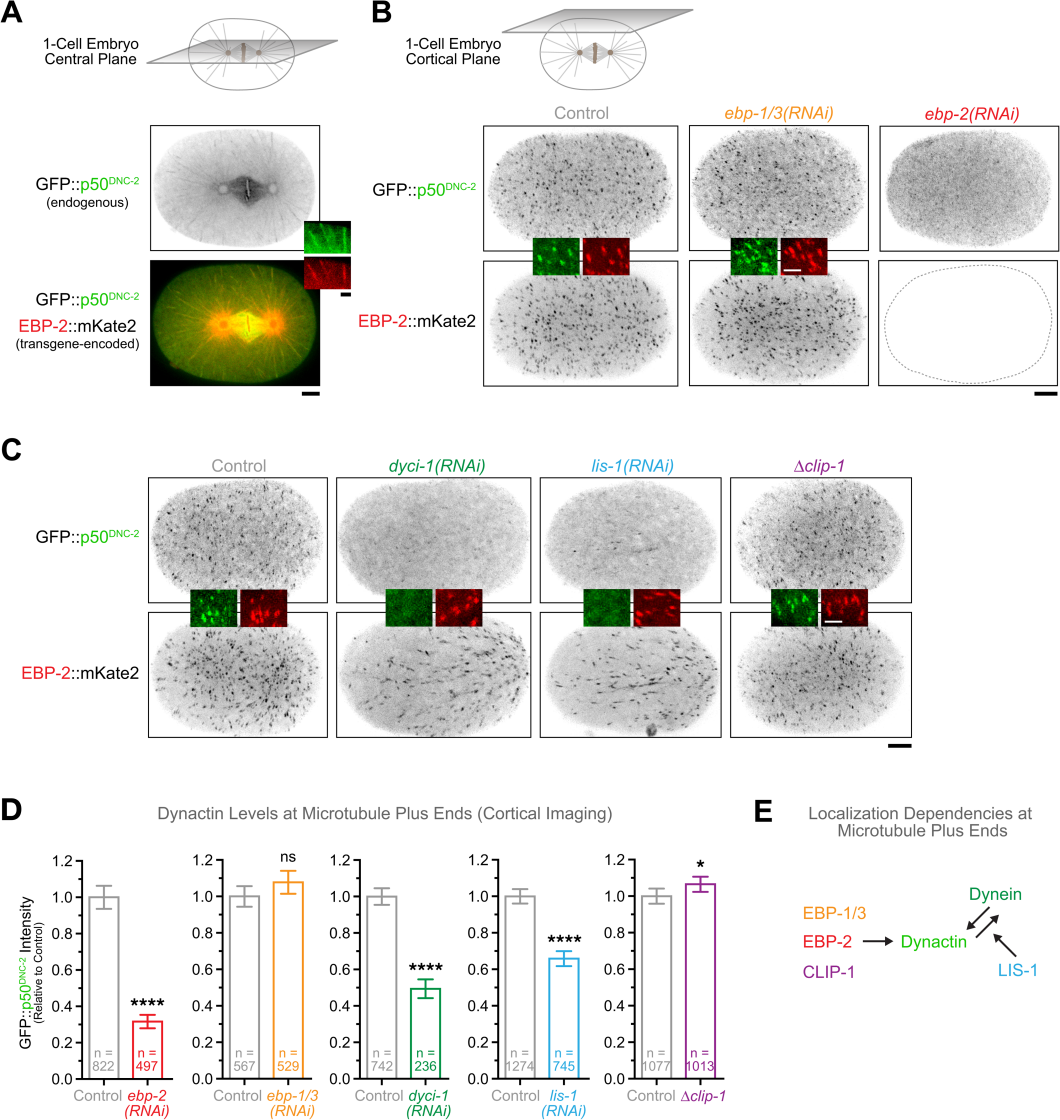
Identification of +TIPs required for MT plus-end targeting of dynein-dynactin in the *C*. *elegans* early embryo. **(A)** Central confocal section in a *C*. *elegans* one-cell embryo at metaphase co-expressing GFP::p50^DNC-2^ and EBP-2::mKate2, showing dynactin enrichment at growing MT plus-ends, kinetochores, and the spindle. Image corresponds to a maximum intensity projection over time (10 frames acquired every 200 ms). Scale bar, 5 μm; insets, 2 μm. **(B)** Cortical confocal section in one-cell embryos co-expressing GFP::p50^DNC-2^ and EBP-2::mKate2, showing that depletion of EBP-2 displaces dynactin from MT plus ends. Images correspond to maximum intensity projections over time (12 frames acquired every 5 s). Scale bar, 5 μm; insets, 2 μm. **(C)** Cortical confocal section in one-cell embryos co-expressing GFP::p50^DNC-2^ and EBP-2::mKate2, showing that depletion of dynein intermediate chain^DYCI-1^ or LIS-1 decreases dynactin levels at MT plus ends. Images correspond to maximum intensity projections over time (12 frames acquired every 5 s). Scale bar, 5 μm; insets, 2 μm. **(D)** Quantification of dynactin levels at MT plus ends using fluorescence intensity measurements of GFP::p50^DNC-2^ at the cortex in the conditions shown in *(B)* and *(C)*. For DYCI-1 and LIS-1 partial depletions were performed (shorter RNAi treatment, see methods), because penetrant depletions resulted in sterility of the mother. Error bars represent the SEM with a 95% confidence interval. For each condition, *n* indicates the total number of individual measurements from 7–11 embryos. The t-test was used to determine statistical significance. ****P < 0.0001; *P < 0.05; ns = not significant, *P* > 0.05. **(E)** Requirements for MT tip tracking by dynein-dynactin in the early *C*. *elegans* embryo.

To investigate whether dynactin's MT binding activity contributes to dynein function in the early *C*. *elegans* embryo, we first asked whether dynactin is present at MT plus ends at this developmental stage. Live confocal imaging in the central plane of metaphase one-cell embryos co-expressing endogenous GFP::p50^DNC-2^ and transgene-encoded EBP-2::mKate2 revealed that dynactin travelled on growing MT tips from mitotic spindle poles to the cell cortex (Fig 1A, S1 Movie). Imaging of the cortical plane allowed end-on visualization of MT tips as they arrived at the cortex (Fig 1B and 1C), which facilitated quantification of dynactin levels at plus ends. Measurements of fluorescence intensity revealed the expected positive correlation between GFP::p50^DNC-2^ and EBP-2::mKate2 levels, but also showed that there is considerable variation in the amount of GFP::p50^DNC-2^ at MT plus ends (S1A Fig). Cortical residency times for EBP-2::mKate2 and GFP::p50^DNC-2^ were nearly identical (1.67 ± 0.03 s and 1.50 ± 0.05 s, respectively) and agreed with previously published measurements for cortical residency times of GFP::EBP-2 (S1C and S1D Fig) [50]. We also generated a *dynein heavy chain*^*dhc-1*^::*gfp* knock-in allele to assess the localization of endogenous dynein. DHC-1::GFP was readily detectable on growing MT plus ends in early embryos (S1B Fig, S2A Fig, S5A Fig, S2 Movie), although the signal appeared weaker than that of GFP::p50^DNC-2^. We conclude that a pool of dynein-dynactin tracks with growing MT plus ends in the early *C*. *elegans* embryo.

We used the quantitative cortical imaging assay to determine which +TIPs were required for MT tip targeting of dynactin and dynein. RNAi-mediated depletion of the three EB paralogs revealed that EBP-2 is required for GFP::p50^DNC-2^ targeting to MT tips, while EBP-1 and EBP-3 are dispensable (Fig 1B and 1D, S2C Fig). In mammalian cells, CLIP-170 acts as an essential linker between EB and dynactin [51–53]. To assess whether the CLIP-170-like protein CLIP-1 recruits dynactin to MT tips in *C*. *elegans*, we generated a null allele of *clip-1* in the *gfp*::*p50*^*dnc-2*^ background (S2D Fig). This revealed that CLIP-1 is dispensable for MT tip localization of GFP::p50^DNC-2^ (Fig 1C and 1D), suggesting that dynactin is directly recruited by EBP-2. Next, we depleted dynein intermediate chain^DYCI-1^ and the dynein co-factor LIS-1. In both cases, GFP::p50^DNC-2^ levels at MT tips decreased substantially (Fig 1C and 1D). Conversely, depletion of p150^DNC-1^ showed that DHC-1::GFP targeting to MT tips was dependent on dynactin (S2A and S2B Fig).

We conclude that in the *C*. *elegans* early embryo, dynein and dynactin are interdependent for targeting to growing MT plus ends and require EBP-2 and LIS-1, but not EBP-1, EBP-3, or the CLIP-170 homolog CLIP-1 (Fig 1E).

### Splice isoforms of the p150^DNC-1^ basic patch modulate microtubule plus-end targeting of dynactin

**Fig 2:**
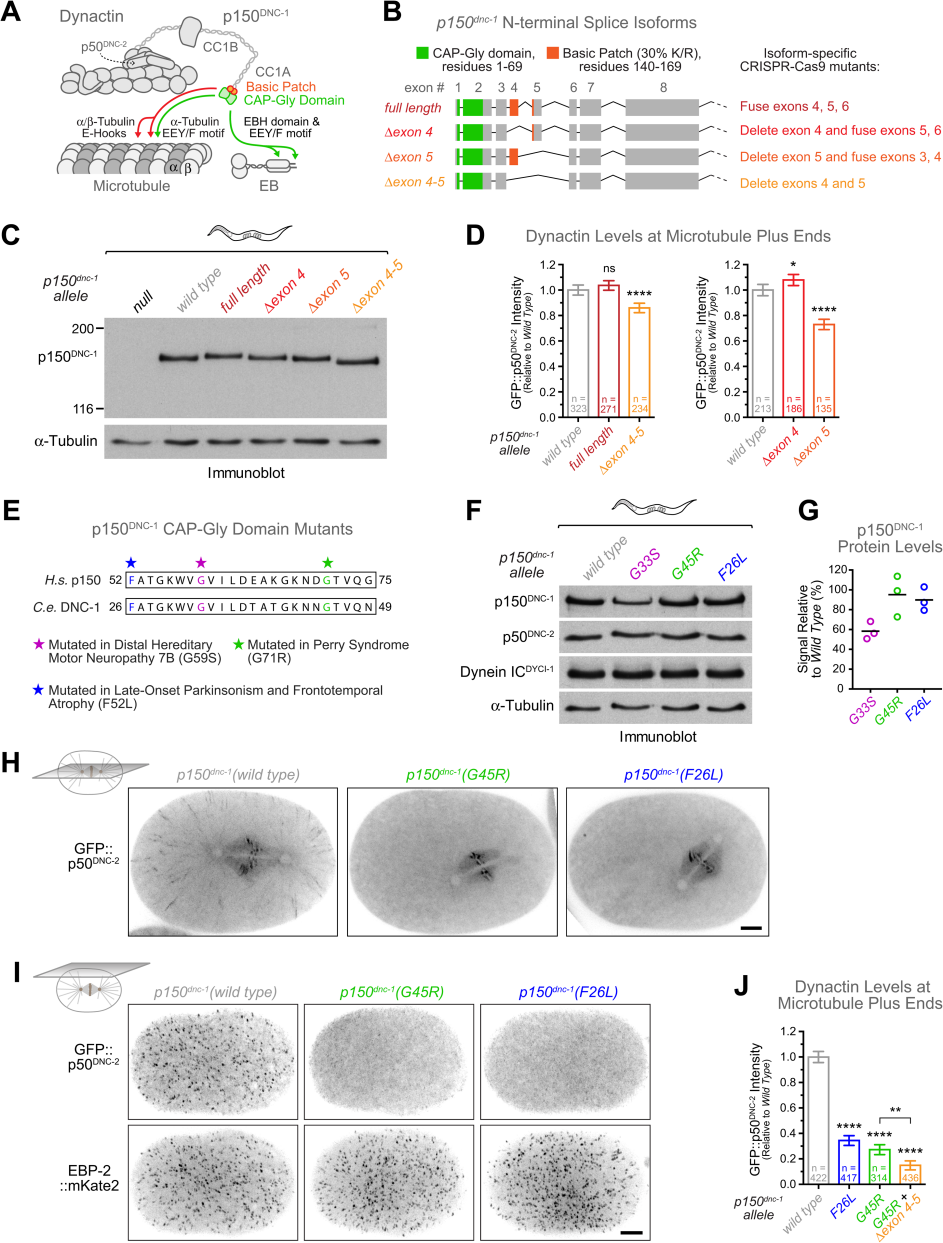
Engineering of p150^DNC-1^ mutants for functional characterization of dynactin's MT binding activity. **(A)** Cartoon of the dynactin complex and its interaction with MTs and end-binding protein (EB). The N-terminal region of the p150^DNC-1^ subunit contains a conserved CAP-Gly domain (residues 1–69) predicted to bind the end-binding homology (EBH) domain of EB and the EEY/F motifs present at the C-termini of EB and α-tubulin. It also contains a basic patch (residues 140–169) predicted to interact with the negatively charged C-terminal tails (E-Hooks) of α- and β-tubulin. **(B)** Schematic of the four *p150*^*dnc-1*^ N-terminal splice isoforms identified by reverse transcription PCR and strategy for CRISPR-Cas9-based genome editing to restrict *p150*^*dnc-1*^ expression to single isoforms. **(C)** Immunoblot of adult worms with an antibody against a C-terminal region of p150^DNC-1^, showing that engineered *p150*^*dnc-1*^ mutants express single isoforms that are distinguishable by size. α-Tubulin was used as the loading control. Molecular mass is indicated in kilodaltons. **(D)** Quantification of GFP::p50^DNC-2^ levels at MT plus ends in *p150*^*dnc-1*^ isoform mutants using fluorescence intensity measurements at the cortex. Error bars represent the SEM with a 95% confidence interval, and *n* indicates the total number of individual measurements from 5–8 different embryos per condition. Statistical significance was determined by one-way ANOVA followed by Bonferroni's multiple comparison test. ****P < 0.0001; *P < 0.05; ns = not significant, *P* > 0.05. **(E)** Sequence alignment of the p150 CAP-Gly domain region where point mutations have been identified that cause neurodegenerative disease in humans. Analogous point mutations were introduced into the *C*. *elegans p150*^*dnc-1*^ locus using CRISPR-Cas9-based genome editing. **(F)** Immunoblots of adult worms, showing that protein levels of p150^DNC-1^ are decreased in the *G33S* mutant but not in the *G45R* and *F26L* mutants. α-Tubulin served as the loading control. **(G)** Quantification of p150^DNC-1^ protein levels using intensity measurements from immunoblots as in *(F)*. Three independent immunoblots were performed for each condition. **(H)** Central confocal section in metaphase one-cell embryos expressing GFP::p50^DNC-2^ and wild-type p150^DNC-1^ or p150^DNC-1^ CAP-Gly mutants. Images correspond to maximum intensity projections over time (10 frames acquired every 200 ms). Scale bar, 5 μm. **(I)** Cortical confocal section of embryos as in *(H)*, additionally expressing EBP-2::mKate2 as a marker for MT plus ends. Images correspond to maximum intensity projections over time (12 images acquired every 5 s). Scale bar, 5 μm. **(J)** Quantification of dynactin levels at MT plus ends using fluorescence intensity measurements of GFP::p50^DNC-2^ at the cortex, as shown in *(I)*. Error bars represent the SEM with a 95% confidence interval, and *n* indicates the number of individual measurements from 8 different embryos per condition. Statistical significance was determined by one-way ANOVA followed by Bonferroni's multiple comparison test. ****P < 0.0001; **P < 0.01.

Having established that dynein and dynactin require the EB homolog EBP-2 for targeting to MT tips, we next examined the role of the dynactin subunit p150^DNC-1^, whose N-terminal CAP-Gly domain (residues 1–69) mediates binding to EB and MTs (Fig 2A). In addition, p150^DNC-1^ contains a ~200-residue basic-serine rich region between the CAP-Gly domain and the first coiled-coil (CC1A), which has been proposed to regulate p150^DNC-1^ association with MTs [54,55] (Fig 2A). The highest density of basic residues is found between residues 140–169 (30% K or R, pI = 12.02). This region is encoded by exon 4 and part of exon 5, which are subject to alternative splicing (Fig 2B, S3A Fig). This is similar to human p150, which contains an alternatively-spliced basic patch of 28 residues (43% K or R, pI = 12.7) adjacent to the CAP-Gly domain [43]. We detected four splice isoforms of *p150*^*dnc-1*^ by reverse transcription PCR of RNA isolated from adult animals (S3B Fig): *full-length p150*^*dnc-1*^ including exons 4 and 5, *p150*^*dnc-1*^ without exon 4 (Δ*exon 4*), *p150*^*dnc-1*^ without exon 5 (Δ*exon 5*), and *p150*^*dnc-1*^ lacking exons 4 and 5 (Δ*exon 4–5*). To define the function of individual splice isoforms, we edited the *p150*^*dnc-1*^ locus to generate animals in which *p150*^*dnc-1*^ expression was restricted to one of the four isoforms (Fig 2B, S3A Fig). Reverse transcription PCR confirmed that animals expressed single *p150*^*dnc-1*^ isoforms corresponding to *full length*, Δ*exon 4*, Δ*exon 5*, or Δ*exon 4–5* (S3B Fig). All mutant animals were homozygous viable and fertile (S3C Fig), demonstrating that none of the p150^DNC-1^ isoforms is essential. Despite differences in predicted molecular weight of only a few kDa (S3A Fig), single isoforms expressed in mutant animals were distinguishable by size on immunoblots with an antibody raised against a C-terminal region of p150^DNC-1^ (Fig 2C). Side-by-side comparison of isoform mutants and wild-type animals on the same immunoblot revealed that neither full-length p150^DNC-1^ nor p150^DNC-1^(Δexon 4–5) is prevalent in wild-type adults (Fig 2C). Instead, immunoblotting, together with reverse transcription PCR data (S3B and S3D Fig), suggested that p150^DNC-1^(Δexon 4) is the predominant isoform.

Humans express the neuron-specific splice variant p135, which lacks the entire N-terminal MT binding region [56]. In *C*. *elegans* hermaphrodite adults, 302 out of 959 somatic cells are neurons, yet we did not find evidence for a *p135*^*dnc-1*^ isoform at the mRNA level (S3D Fig), nor did our p150^DNC-1^ antibody detect any protein below ~150 kDa in wild-type animals (Fig 2C). We also generated a *p150*^*dnc-1*^::*3xflag* knock-in allele, and immunoblotting with antibody against 3xFLAG similarly failed to detect a p135 isoform (S3E Fig). We speculated that specifically suppressing the expression of p150^DNC-1^ isoforms might facilitate the detection of p135 and engineered a null allele of *p150*^*dnc-1*^ by inserting a stop codon in exon 1 immediately following the start codon (S3A Fig). The null mutation did not affect splicing of *p150*^*dnc-1*^ mRNA (S3B Fig) and therefore should permit expression of p135 from an alternative start codon, as is the case in humans [56]. However, immunoblotting produced no evidence of p135 expression in the absence of p150^DNC-1^ isoforms (Fig 2C). We conclude that *C*. *elegans* does not express significant amounts of a p135 isoform.

Next, we used cortical imaging of GFP::p50^DNC-2^ in one-cell embryos to determine the effect of p150^DNC-1^ isoforms on dynactin recruitment to MT tips. Full-length p150^DNC-1^ and p150^DNC-1^(Δexon 4) fully supported dynactin targeting to MT tips, and dynactin levels were even slightly increased (108 ± 4% of controls) for the Δexon 4 isoform (Fig 2D). By contrast, expression of p150^DNC-1^(Δexon 5) or p150^DNC-1^(Δexon 4–5) decreased dynactin levels at MT tips to 73 ± 4% and 86 ± 4% of controls, respectively (Fig 2D). Thus, surprisingly, the similarly basic regions encoded by exon 4 (27% K/R; pI = 11.2) and exon 5 (19% K/R; pI = 12) make differential contributions to dynactin targeting. We conclude that splice isoforms of p150^DNC-1^ regulate dynactin levels at MT tips.

### Point mutations in p150^DNC-1^'s CAP-Gly domain that cause neurodegenerative disease in humans displace dynactin and dynein from microtubule plus ends

Our analysis of p150^DNC-1^ isoforms suggested that the basic region had a relatively minor role in targeting dynactin to MT tips. To examine the role of the CAP-Gly domain, we used genome editing to separately introduce three point mutations into p150^DNC-1^ that compromise CAP-Gly domain function and cause neurodegenerative disease in humans (Fig 2E): G33S corresponds to human G59S, which causes motor neuropathy 7B [27]; G45R corresponds to human G71R, which causes Perry Syndrome [28]; and F26L corresponds to human F52L, which was recently identified in a patient with Perry Syndrome-like symptoms [29]. The *F26L* and *G45R* mutants could be propagated as homozygotes with high embryonic viability (99 ± 1% and 90 ± 2%, respectively), whereas the *G33S* mutant was lethal in the F2 generation (1 ± 1% embryonic viability) (S4A Fig). Immunoblotting of homozygous F1 adults showed that *G33S* animals had decreased levels of p150^DNC-1^, indicating that the mutation destabilized the protein (Fig 2F and 2G). By contrast, total levels of p150^DNC-1^ were not affected in the *F26L* or *G45R* mutant. Central plane imaging in one-cell embryos expressing GFP::p50^DNC-2^ showed that dynactin containing the F26L or G45R mutation was present on the mitotic spindle and prometaphase kinetochores but displaced from MT tips (Fig 2H, S3 Movie). Cortical imaging after introduction of the EBP-2::mKate2 marker revealed that GFP::p50^DNC-2^ levels at MT tips were reduced to 34 ± 4% and 27 ± 4% of controls in the *F26L* and *G45R* mutant, respectively (Fig 2I and 2J, S4 Movie, S5 Movie). Deletion of the basic patch encoded by exons 4 and 5 in the *G45R* mutant (*G45R +* Δ*exon 4–5*) further reduced GFP::p50^DNC-2^ levels at MT tips to 15 ± 4% (Fig 2J) but had no additive effect on embryonic viability (90 ± 2%) (S4A Fig). Additional quantifications showed that in both the *F26L* and *G45R* mutant, GFP::p50^DNC-2^ still targeted to the nuclear envelope and kinetochores, while GFP::p50^DNC-2^ levels were reduced on spindle MTs (S4B Fig). We also introduced the mutations into animals expressing DHC-1::GFP, which confirmed that dynein levels were decreased at MT tips and on spindle MTs (S5B–S5D Fig). We conclude that point mutations in the p150^DNC-1^ CAP-Gly domain that cause human neurodegenerative disease reduce dynein-dynactin levels on MTs and greatly diminish the ability of dynein-dynactin to track with MT tips.

### *p150*^*dnc-1*^ CAP-Gly mutants exhibit defects in centration/rotation of the nucleus-centrosome complex, chromosome congression, and spindle rocking

**Fig 3:**
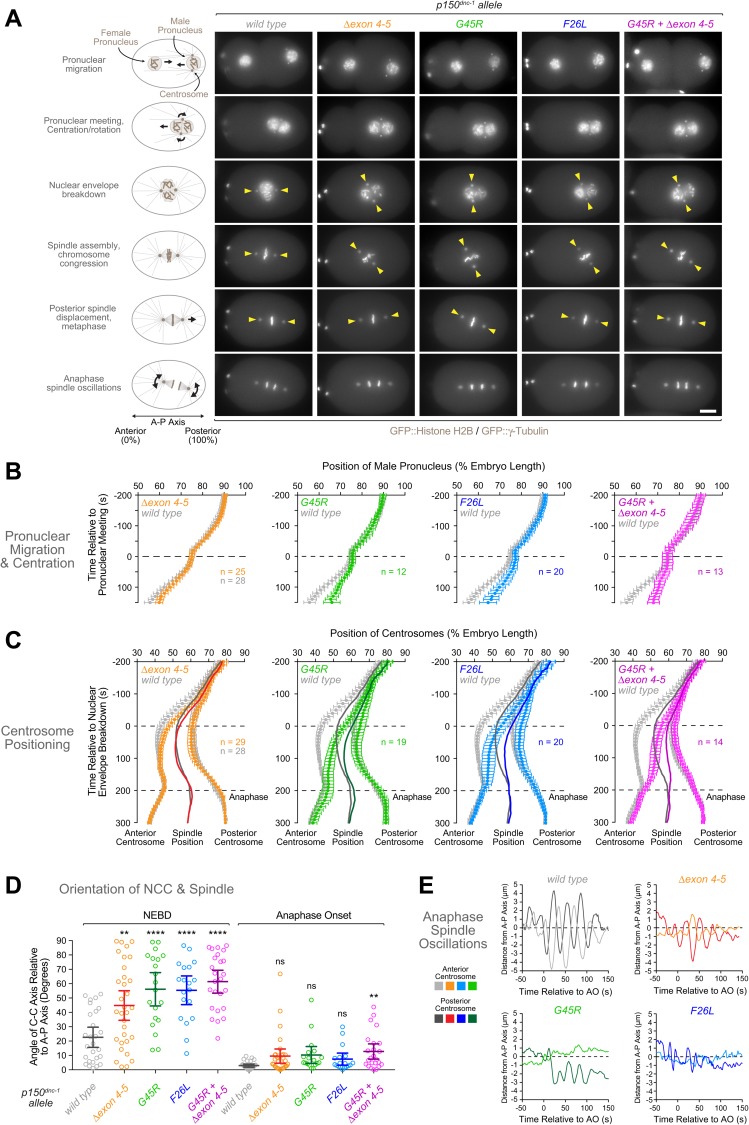
Mutations in the p150^DNC-1^ CAP-Gly domain and basic region cause defects in centration/rotation of the nucleus-centrosome complex. **(A)** Selected frames from time-lapse sequences of the first embryonic division in controls and *p150*^*dnc-1*^ mutants. Chromosomes and centrosomes are marked with GFP::histone H2B and GFP::γ-tubulin, respectively. Scale bar, 5 μm. **(B)** Migration kinetics of the male pronucleus in the embryos shown in *(A)*. The position of the male pronucleus along the anterior-posterior axis was determined in images captured every 10 s. Individual traces were normalized to embryo length, time-aligned relative to pronuclear meeting, averaged for the indicated number (n) of embryos, and plotted against time. Error bars represent the SEM with a 95% confidence interval. **(C)** Kinetics of centrosome positioning along the anterior-posterior axis, determined as described for *(B)* and plotted relative to nuclear envelope breakdown. Solid lines indicate the midpoint between the two centrosomes (spindle position). Anaphase begins at 200 s. Error bars represent the SEM with a 95% confidence interval. **(D)** Angle between the centrosome-centrosome (C-C) axis and the anterior-posterior (A-P) axis in one-cell embryos at nuclear envelope breakdown (NEBD) and anaphase onset. Circles correspond to measurements in individual embryos. Error bars represent the SEM with a 95% confidence interval. Statistical significance was determined by one-way ANOVA followed by Bonferroni's multiple comparison test. ****P < 0.0001; **P < 0.01; ns = not significant, *P* > 0.05. **(E)** Representative examples of spindle oscillations in anaphase. The transverse position of spindle poles was determined every 2 s and plotted against time.

Next, we asked whether the *p150*^*dnc-1*^ mutants affected dynein-dynactin function in the one-cell embryo. We crossed the mutants with animals co-expressing GFP::histone H2B and GFP::γ-tubulin, which allowed precise tracking of pronuclei and centrosomes, respectively (Fig 3A). None of the mutants exhibited defects in centrosome separation, and pronuclear migration along the anterior-posterior axis proceeded with normal kinetics until pronuclear meeting, which occurred at the correct position in the posterior half of the embryo (Fig 3A and 3B, S6A Fig). However, subsequent centration of the nucleus-centrosome complex (NCC) slowed substantially in *p150*^*dnc-1*^ *F26L*, *G45R*, and *G45R +* Δ*exon 4–5* mutants, and NCC rotation was defective (Fig 3A–3D, S6 Movie). NCC centration was not significantly perturbed in the isoform mutants (S6A and S6B Fig), but the Δ*exon 4–5* mutant exhibited defects in NCC rotation (Fig 3D, S6C Fig). In all mutants, spindle orientation recovered during prometaphase, so that the spindle axis was largely aligned with the anterior-posterior axis of the embryo at the time of anaphase onset (Fig 3D, S6C Fig).

In controls, the mitotic spindle was displaced from the embryo center toward the posterior in preparation for asymmetric division (Fig 3C). By contrast, spindle assembly in *p150*^*dnc-1*^ CAP-Gly mutants already occurred in the posterior half of the embryo, and the spindle had to be moved only slightly to the posterior to be correctly positioned. In controls, the regular and vigorous oscillations of spindle rocking began at anaphase onset and lasted for approximately 100 s (Fig 3E, S7 Movie). By contrast, spindle rocking in *p150*^*dnc-1*^ CAP-Gly mutants was irregular and significantly dampened.

In addition to defects in NCC centration/rotation and spindle rocking, we observed a slight but consistent delay in chromosome congression in *p150*^*dnc-1*^ CAP-Gly mutants, indicating problems with the interactions between chromosomes and spindle MTs (S7A and S7B Fig, S6 Movie, S7 Movie). This did not result in obvious chromosome mis-segregation in the first embryonic division (S7A Fig). However, when the spindle assembly checkpoint (SAC) was inactivated by RNA-mediated depletion of Mad1^MDF-1^, embryonic viability decreased by 28% and 22% in the *G45R* and *F26L* mutant, respectively, whereas *Mad1*^*mdf-1*^*(RNAi)* in controls decreased embryonic viability by just 6% (S7C Fig). This suggests that SAC signaling is required during embryogenesis to prevent chromosome segregation errors when the p150^DNC-1^ CAP-Gly domain is compromised.

We conclude that mutations in the p150^DNC-1^ CAP-Gly domain perturb a specific subset of dynein-dynactin functions in the one-cell embryo.

### Inhibition of cortical dynein and *p150*^*dnc-1*^ CAP-Gly mutants cause distinct defects during centration of the nucleus-centrosome complex

**Fig 4:**
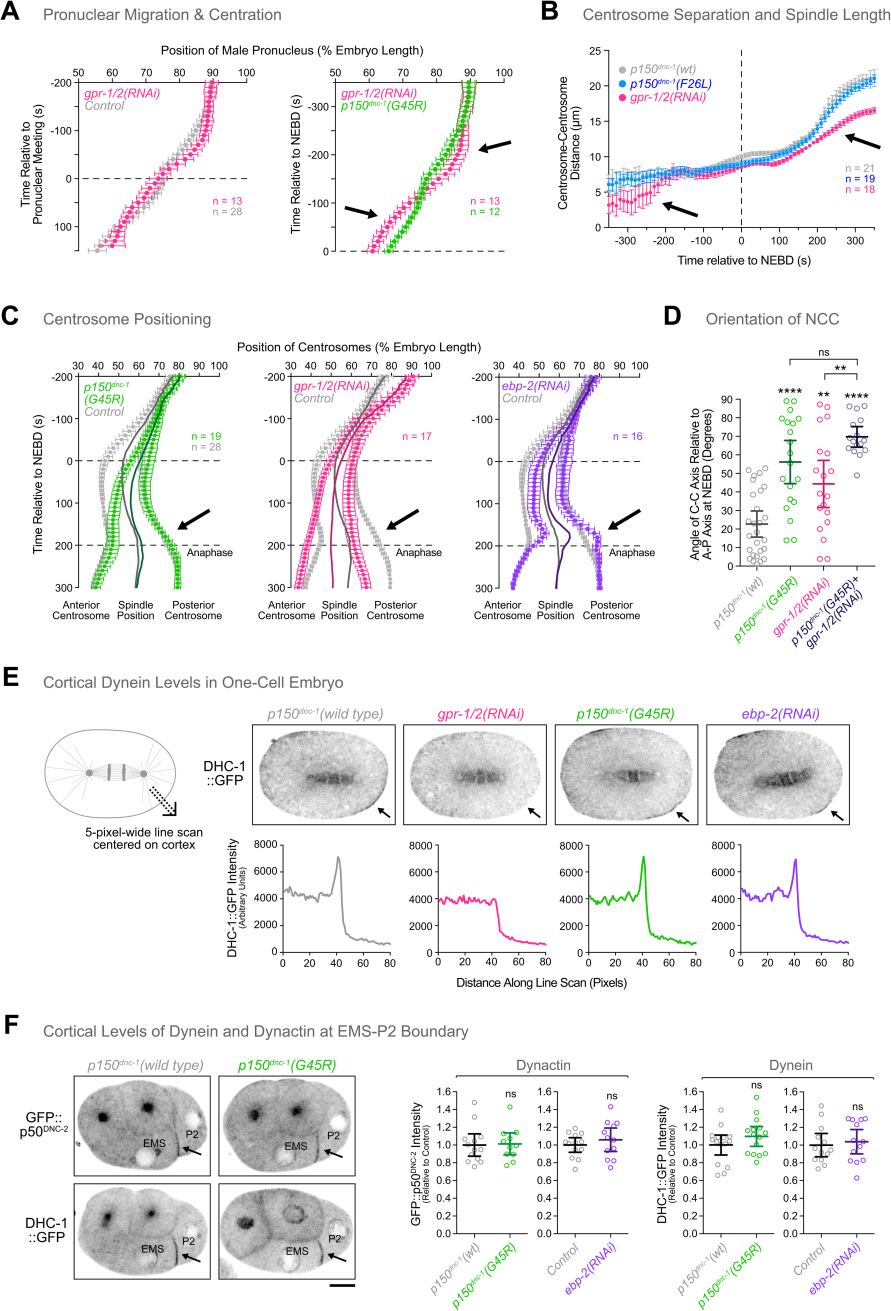
MT tip tracking of dynein-dynactin is not required for targeting the motor to the cell cortex, nor for the generation of robust cortical pulling forces. **(A)** Migration kinetics of the male pronucleus in one-cell embryos, showing that inhibition of cortical pulling forces and *p150*^*dnc-1*^ CAP-Gly mutants cause distinct defects in centration. The position of the male pronucleus, marked by GFP::histone H2B, was determined along the anterior-posterior axis in images captured every 10 s. Individual traces were normalized to embryo length, time-aligned relative to pronuclear meeting *(left)* or nuclear envelope breakdown (NEBD) *(right)*, averaged for the indicated number (n) of embryos, and plotted against time. Arrows point to differences in migration kinetics between the *p150*^*dnc-1*^*(G45R)* mutant and *gpr-1/2(RNAi)*. Error bars represent the SEM with a 95% confidence interval. **(B)** Plot of centrosome-centrosome distance over time in one-cell embryos expressing GFP::γ-tubulin. Measurements were made in images captured every 10 s. Individual traces were time-aligned relative to NEBD, averaged for the indicated number (n) of embryos, and plotted against time. Error bars represent the SEM with a 95% confidence interval. Arrows points to delays in centrosome separation (-300 s) and to defective mitotic spindle elongation (200 s) in embryos depleted of GPR-1/2. **(C)** Positioning of centrosomes, marked by GFP::γ-tubulin, measured in time-lapse sequences as described for *(A)* and plotted relative to NEBD. Solid lines indicate the midpoint between the two centrosomes (spindle position). Error bars represent the SEM with a 95% confidence interval. Arrows highlight the difference in posterior centrosome displacement between the *p150*^*dnc-1*^*(G45R)* mutant, *gpr-1/2(RNAi)*, and *ebp-2(RNAi)*. **(D)** Angle between the centrosome-centrosome (C-C) axis and the anterior-posterior (A-P) axis in one-cell embryos at NEBD. Circles correspond to measurements in individual embryos. Error bars represent the SEM with a 95% confidence interval. Statistical significance was determined by one-way ANOVA followed by Bonferroni's multiple comparison test. ****P < 0.0001; **P < 0.01; ns = not significant, *P* > 0.05. **(E)** *(top)* Central confocal section in one-cell embryos at anaphase expressing dynein heavy chain^DHC-1^::GFP, showing that cortical enrichment of dynein (arrows) is unperturbed in the *p150*^*dnc-1*^*(G45R)* mutant and after depletion of EBP-2. *(bottom)* Line scans (5 pixels wide, 80 pixels long) were drawn across the cortex as indicated in the schematic on the left, and DHC-1::GFP raw intensity was plotted against the position along the line scan. **(F)** *(left)* Stills from a time-lapse sequence in 4-cell embryos expressing GFP::p50^DNC-2^ *(top)* or dynein heavy chain^DHC-1^::GFP *(bottom)*, showing normal accumulation of dynein-dynactin at the EMS-P2 cell border in the *p150*^*dnc-1*^*(G45R)* mutant and after depletion of EBP-2. Scale bar, 5 μm. *(right)* Quantification of dynein and dynactin levels at the EMS-P2 cell border using fluorescence intensity measurements. Error bars represent the SEM with a 95% confidence interval. The t-test was used to determine statistical significance. ns = not significant, *P* > 0.05.

Anaphase spindle rocking requires cortical dynein pulling on astral MTs [2,57]. Since spindle rocking was affected in *p150*^*dnc-1*^ CAP-Gly mutants, we sought to assess the extent of phenotypic overlap between *p150*^*dnc-1*^ CAP-Gly mutants and inhibition of dynein-dependent cortical pulling. We tracked centrosomes and pronuclei after co-depleting GPR-1 and GPR-2, which are required for cortical anchoring of dynein-dynactin [2,57]. In contrast to *p150*^*dnc-1*^ CAP-Gly mutants, *gpr-1/2(RNAi)* delayed the initial separation of centrosomes and the onset of pronuclear migration (Fig 4A–4C). Pronuclear migration and NCC centration subsequently occurred at a slightly faster rate than in controls, so that the NCC achieved near-normal centration by nuclear envelope breakdown (NEBD) (Fig 4A and 4C). These results are consistent with slowed centrosome separation and faster centering reported after co-depletion of GOA-1 and GPA-16, the Gα proteins acting upstream of GPR-1/2 [58,59]. Thus, the kinetics of pronuclear migration and NCC centration differ between *gpr-1/2(RNAi)* and *p150*^*dnc-1*^ CAP-Gly mutants. NCC rotation, by contrast, was affected in both perturbations (Fig 4D). Importantly, *gpr-1/2(RNAi)* in the *p150*^*dnc-1*^*(G45R)* mutant enhanced the rotation defect of *gpr-1/2(RNAi)* on its own, arguing that GPR-1/2 and the p150^DNC-1^ CAP-Gly domain contribute to NCC rotation through parallel pathways. After NEBD, depletion of GPR-1/2 prevented posterior displacement of the spindle and the lack of cortical pulling was especially evident in the track of the posterior centrosome (Fig 4C). In addition, the mitotic spindle was shorter than controls during metaphase and failed to elongate properly in anaphase (Fig 4B). By contrast, posterior centrosome movement towards the cortex in *p150*^*dnc-1*^ CAP-Gly mutants was indistinguishable from controls (Fig 3C, Fig 4C), and spindle length was normal throughout metaphase and anaphase (Fig 4B). These results argue that, although spindle rocking is compromised in *p150*^*dnc-1*^ CAP-Gly mutants, cortical dynein is still able to generate robust pulling forces on astral MTs. We also tracked centrosomes after depletion of EBP-2, which, just like *p150*^*dnc-1*^ CAP-Gly mutants, delocalized dynein-dynactin from MT tips (Fig 1B and 1D). Strikingly, posterior spindle displacement was exaggerated in *ebp-2(RNAi*) embryos compared with controls (Fig 4C). These results suggest that cortical pulling forces used for asymmetric spindle positioning can be generated in the absence of MT tip-localized dynein-dynactin.

### MT plus-end tracking of dynein-dynactin is dispensable for targeting the motor to the cell cortex

The robust dynein-dependent cortical pulling observed in one-cell embryos depleted of EBP-2 and in embryos of *p150*^*dnc-1*^ CAP-Gly mutants implied that the motor was able to target to the cortex under these conditions. To test this directly, we measured the intensity of the DHC-1::GFP signal in line scans drawn across the cortex in one-cell embryos at anaphase. This revealed a cortically enriched pool of DHC-1::GFP that was dependent on GPR-1/2, as expected (Fig 4E). Cortical dynein in the one-cell embryo was unaffected in the *p150*^*dnc-1*^*(G45R)* mutant and after *ebp-2(RNAi)*. We also imaged the 4-cell embryo, in which dynactin and dynein become prominently enriched at the EMS-P2 cell border prior to EMS and P2 spindle rotation [60,61]. Quantification of GFP::p50^DNC-2^ and DHC-1::GFP levels at the EMS-P2 cell border revealed that cortical levels of dynein-dynactin were unchanged in the *p150*^*dnc-1*^*(G45R)* mutant and after *ebp-2(RNAi)* (Fig 4F). We conclude that MT tip tracking of dynein-dynactin is dispensable for cortical targeting of the motor in the early embryo.

### Mutation of α-tubulin's C-terminal tyrosine causes defects in NCC centration/rotation that resemble those of *p150*^*dnc-1*^ CAP-Gly mutants

**Fig 5:**
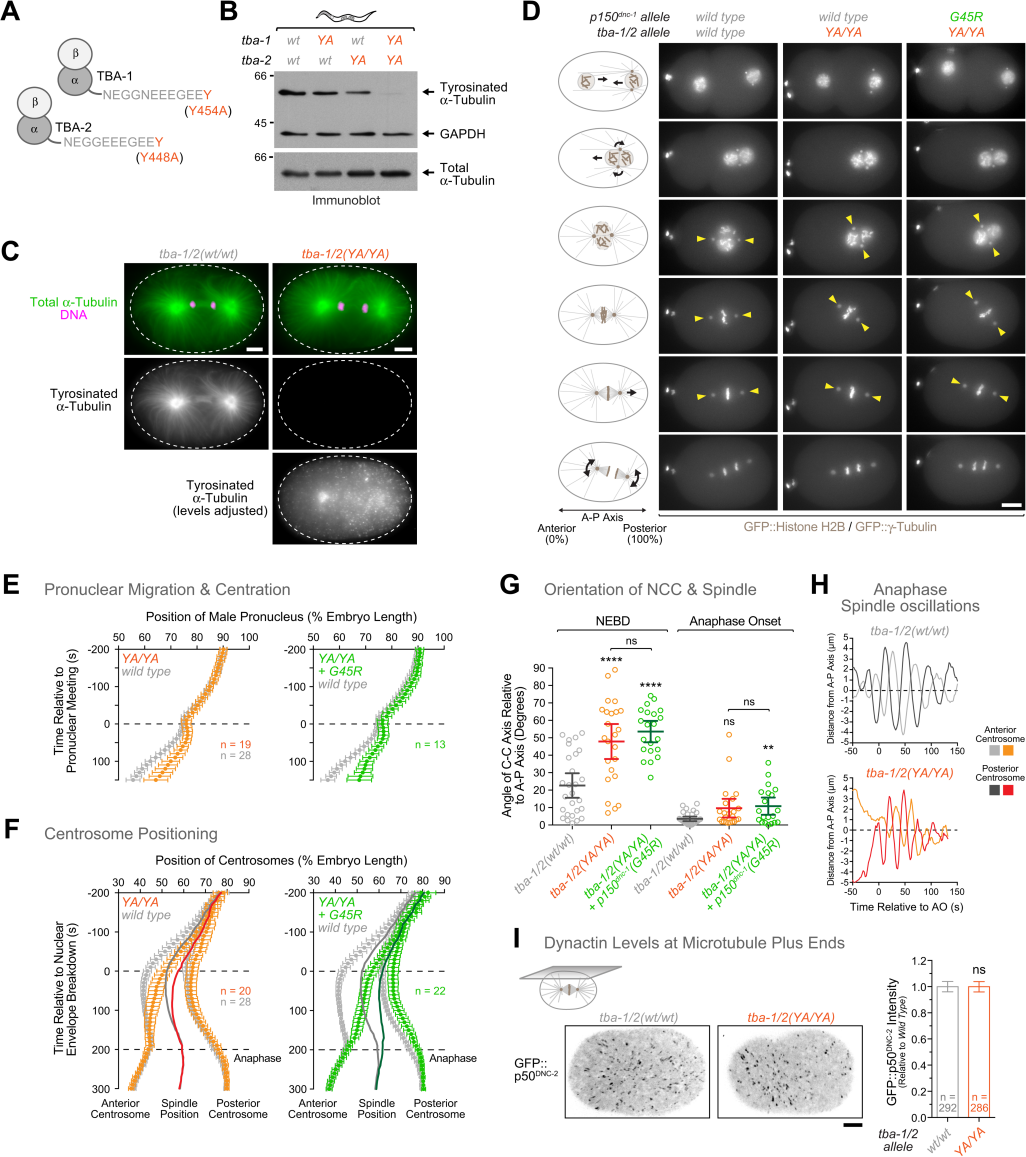
The C-terminal tyrosine of α-tubulin is required for centration/rotation of the nucleus-centrosome complex but not for MT tip tracking of dynactin. **(A)** Cartoon depicting the C-terminal tail of TBA-1 and TBA-2, the major α-tubulin isotypes in early embryogenesis. The C-terminal tyrosine residues were mutated to alanine using CRISPR-Cas9-based genome editing. **(B)** Immunoblot of adult worms showing decreased levels of tubulin tyrosination in α-tubulin *YA* mutants. The same membrane was probed sequentially with an antibody against tyrosinated α-tubulin (antibody YL1/2) and against total α-tubulin (antibody B512). GAPDH served as a loading control. Molecular mass is indicated in kilodaltons. **(C)** Immunofluorescence images showing lack of tubulin tyrosination in *tba-1/2(YA/YA)* embryos at the one-cell stage (anaphase). Embryos were co-stained with antibodies against tyrosinated α-tubulin and total α-tubulin. Image with adjusted intensity levels shows unspecific background signal in the α-tubulin tyrosine mutant. Scale bars, 5 μm. **(D)—(H)** Still images from time-lapse sequences *(D)* and analysis of male pronuclear migration *(E)*, centrosome positioning *(F)*, NCC/spindle orientation *(G)*, and anaphase spindle oscillations *(H)* during the first embryonic division for controls and α-tubulin tyrosine mutants. Data is displayed as described for Fig 3. Statistical significance was determined by one-way ANOVA followed by Bonferroni's multiple comparison test. ****P < 0.0001; **P < 0.01; ns = not significant, *P* > 0.05. Scale bar, 5 μm. **(I)** *(left)* Cortical confocal section of a control and *tba-1/2(YA/YA)* one-cell embryo in metaphase expressing GFP::p50^DNC-2^. Images correspond to maximum intensity projections over time (12 images acquired every 5 s). Scale bar, 5 μm. *(right)* Quantification of dynactin levels at MT plus ends using fluorescence intensity measurements of GFP::p50^DNC-2^ at the cortex. Error bars represent the SEM with a 95% confidence interval, and *n* indicates the number of individual measurements from 8–9 different embryos per condition. The t-test was used to determine statistical significance. ns = not significant, *P* > 0.05.

CAP-Gly domains bind the C-terminal EEY/F motif of α-tubulin, and the tyrosine residue is critical for the interaction (Fig 2A) [22]. We therefore asked whether decreased affinity of dynactin for tyrosinated MTs could be contributing to the defects observed in *p150*^*dnc-1*^ CAP-Gly mutants. Of the 9 α-tubulin isoforms in *C*. *elegans*, TBA-1 and TBA-2 are the major α-tubulin isotypes expressed during early embryogenesis [62]. We mutated the C-terminal tyrosine of TBA-1 and TBA-2 to alanine and obtained animals homozygous for either mutation alone (*YA*) or both mutations combined (*YA/YA*) (Fig 5A). Immunoblotting of adult animals with the monoclonal antibody YL1/2, which is specific for tyrosinated tubulin, revealed that levels of tubulin tyrosination were decreased in *tba-1(YA)* and *tba-2(YA)* single mutants, with *tba-2(YA)* having a more pronounced effect (Fig 5B). Combining the two mutations dramatically decreased total levels of tubulin tyrosination. Importantly, immunoblotting with an antibody insensitive to tubulin tyrosination confirmed that total α-tubulin levels were not affected in the three mutants (Fig 5B). We then used immunofluorescence to directly assess tyrosinated tubulin levels in the early embryo. In controls, the mitotic spindle of the one-cell embryo was prominently stained with the antibody against tyrosinated tubulin (Fig 5C). By contrast, the tubulin tyrosination signal was undetectable in the *tba-1/2(YA/YA)* double mutant, despite normal spindle assembly. Thus, we were able to generate animals without detectable tubulin tyrosination in early embryos.

We next addressed the functional significance of tubulin tyrosination in the one-cell embryo. Strikingly, we found that the *tba-1/2(YA/YA)* mutant exhibited NCC centration/rotation defects reminiscent of those observed in *p150*^*dnc-1*^ CAP-Gly mutants (Fig 5D–5G, S8 Movie). Combining the *tba-1/2(YA/YA)* mutant with the *p150*^*dnc-1*^*(G45R)* mutant did not significantly exacerbate the centration/rotation defects of the either mutant on its own, indicating that both mutants act in the same pathway (Fig 5D–5G, S8 Movie). Interestingly, in contrast to *p150*^*dnc-1*^ CAP-Gly mutants, anaphase spindle rocking was not affected in the *tba-1/2(YA/YA)* mutant (Fig 5H).

We also examined the effect of the *tba-1/2(YA/YA)* mutant on GFP::p50^DNC-2^ localization and found that dynactin levels at MT tips were identical to controls (Fig 5I). Thus, in contrast to mouse fibroblasts [25], tubulin tyrosination in the *C*. *elegans* embryo is not required to target dynactin to MT tips.

### *p150*^*dnc-1*^ CAP-Gly mutants and α-tubulin tyrosine mutants decrease the frequency of centrosome-directed early endosome movements

**Fig 6:**
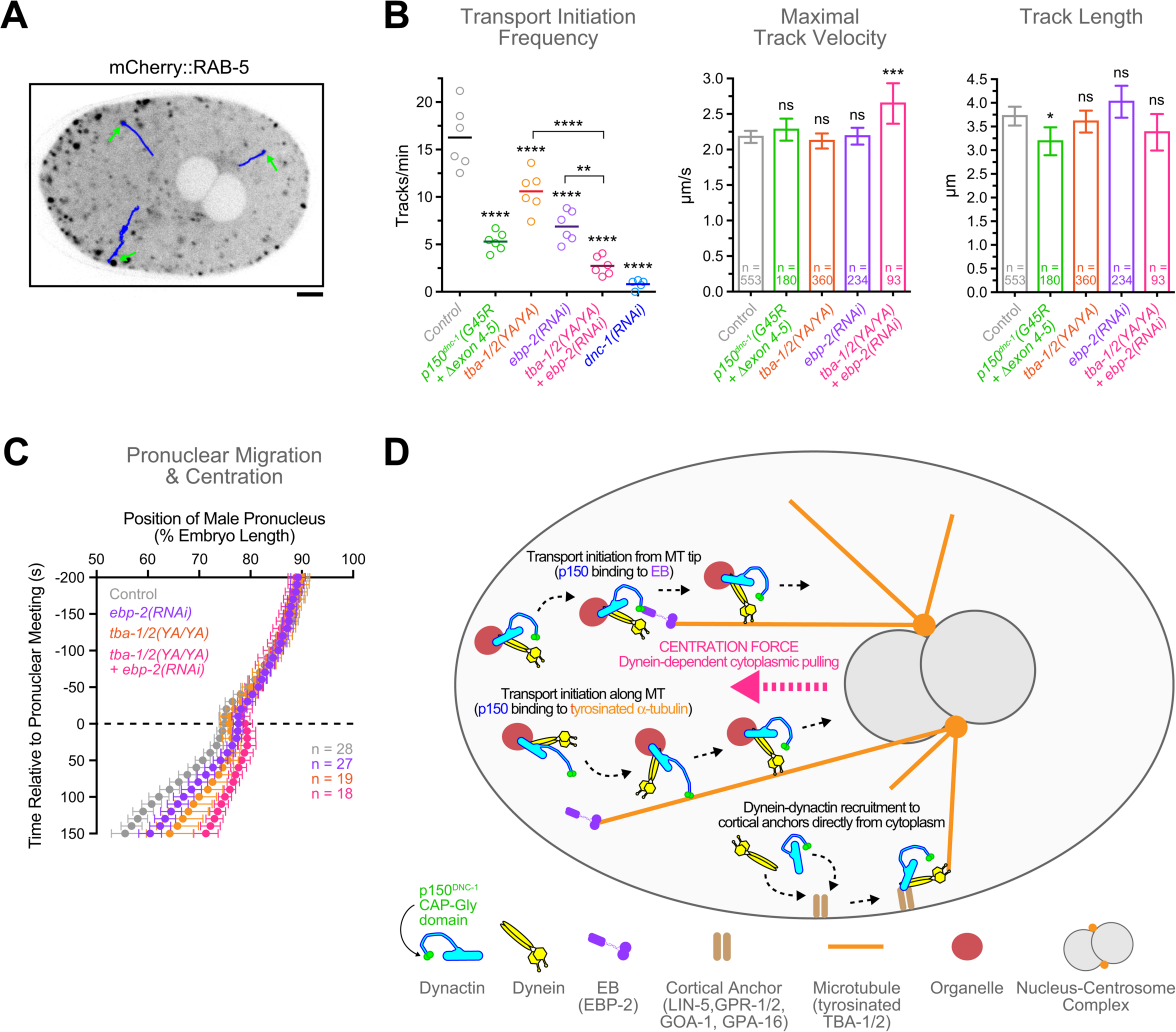
Dynactin's MT binding activity, tyrosinated α-tubulin, and EBP-2 are required for efficient initiation of centrosome-directed organelle transport. **(A)** Selected image from a time-lapse sequence recorded during centration of the nucleus-centrosome complex in an embryo expressing the early endosome marker mCherry::RAB-5. Arrows point to early endosomes that are about to move from the cell periphery towards the centrosomes along the tracks shown in blue. Scale bar, 5 μm. **(B)** Quantification of the number of tracks per min, the maximal track velocities, and the total track displacement for mCherry::RAB-5-labelled particles travelling towards centrosomes during the centration/rotation phase. Particles were tracked in time-lapse sequences as shown in *(A)* with images captured every 400 ms. Circles correspond to data points of individual embryos. Error bars represent the SEM with a 95% confidence interval, and *n* indicates the number of individual tracks analyzed from 8–9 embryos per condition. Statistical significance was determined by one-way ANOVA followed by Bonferroni's multiple comparison test. ****P < 0.0001; ***P < 0.001; **P < 0.01; *P < 0.05; ns = not significant, *P* > 0.05. **(C)** Migration kinetics of the male pronucleus in one-cell embryos, showing that depletion of EBP-2 in the *tba-1/2(YA/YA)* mutant results in enhanced centration defects relative to either perturbation alone. The position of the male pronucleus (marked by GFP::histone H2B as in Figs 3A and 5D) along the anterior-posterior axis was determined in images captured every 10 s. Individual traces were normalized to embryo length, time-aligned relative to pronuclear meeting, averaged for the indicated number (n) of embryos, and plotted against time. Error bars represent the SEM with a 95% confidence interval. **(D)** Model summarizing the functional significance of dynactin's MT binding activity in the one-cell embryo. The CAP-Gly domain of p150^DNC-1^ binds to MTs and MT tips through interactions involving the C-terminal tails of α-tubulin and EBP-2, respectively. This promotes the initiation of organelle transport by dynein along the length of MTs, as well as from MT tips. Dynein-dependent movement of organelles along MTs towards centrosomes generates cytoplasmic pulling forces required for centration of the nucleus-centrosome complex. Tracking of dynein-dynactin with MT tips is not required for the delivery of the motor to cortical anchors. Instead, dynein-dynactin bind to the cortex directly from the cytoplasm and subsequently capture MT tips for generation of cortical pulling forces.

Dynein-mediated transport of small organelles along MTs towards centrosomes is proposed to generate the cytoplasmic pulling forces for centration (the centrosome-organelle mutual pulling model) [5,63,64]. To ask whether the centration defects in our mutants correlate with defects in MT minus end-directed organelle transport, we monitored the movement of early endosomes, labelled with mCherry::RAB-5, from pronuclear meeting until NEBD. Time-lapse sequences recorded in a focal plane that included the NCC were used for semi-automated tracking of early endosomes that moved from the cell periphery towards centrosomes (Fig 6A). In control embryos, we counted 16.3 ± 3.5 tracks/min during the ~6 min centration interval (Fig 6B). This was reduced to 0.8 ± 0.6 tracks/min in embryos depleted of p150^DNC-1^ by RNAi, confirming that dynactin is required for early endosome movement directed towards centrosomes. The *p150*^*dnc-1*^*(G45R +* Δ*exon 4–5)* mutant also strongly reduced the number of observed tracks to 5.3 ± 1.1 tracks/min (Fig 6B, S9 Movie). The *tba-1/2(YA/YA)* mutant had a less severe effect but still substantially reduced the number of tracks to 10.6 ± 2.2 per min. We also determined the maximal velocity in each track (determining the mean speed was complicated by frequent pausing of particles) and the total track displacement. This revealed only minor differences between controls and either mutant (Fig 6B). We conclude that *p150*^*dnc-1*^ CAP-Gly and α-tubulin tyrosine mutants reduce the frequency with which early endosomes move towards centrosomes during the centration phase. These results are consistent with the idea that dynactin binding to tyrosinated MTs enhances the efficiency of transport initiation by dynein, as recently documented *in vitro* [26].

### EBP-2 and tubulin tyrosination make independent contributions to initiation of dynein-mediated organelle transport and NCC centration

In the distal axon of neuronal cells, EB-dependent recruitment of dynactin to dynamic MT plus ends is proposed to ensure efficient initiation of retrograde transport by dynein [32]. To test whether EBP-2 plays a role in the initiation of centrosome-directed organelle transport in the one-cell *C*. *elegans* embryo, we tracked early endosomes after *ebp-2(RNAi)*. The number of early endosome tracks was reduced from 16.3 ± 3.5 to 6.9 ± 1.7 per minute after EBP-2 depletion (Fig 6B). Strikingly, *ebp-2(RNAi)* in the *tba-1/2(YA/YA)* mutant further reduced the number of early endosome tracks to 2.7 ± 1.0 per minute (Fig 6B) and enhanced the NCC centration defect compared to the individual perturbations (Fig 6C, S8 Movie). This suggests that EBP-2 is able to promote the initiation of dynein-mediated transport from MT tips even in the absence of tubulin tyrosination, consistent with the observation that dynactin targeting to MT tips is unaffected in the tubulin tyrosine mutant (Fig 5I). We conclude that EBP-2 and tubulin tyrosination independently contribute to the initiation of dynein-mediated organelle transport and NCC centration.

## Discussion

Dynactin's MT binding activity is crucial in neurons, as illustrated by single point mutations that compromise the function of the p150 CAP-Gly domain and cause neurodegenerative disease [30,31,65,66]. Here, we introduced these CAP-Gly mutations into *C*. *elegans* p150^DNC-1^ to investigate how dynactin's interaction with MTs and +TIPs contributes to dynein function in early embryogenesis. Together with the analysis of engineered *p150*^*dnc-1*^ splice isoform and tubulin tyrosine mutants, our work provides insight into the regulation and function of MT tip tracking by dynein-dynactin in animals and uncovers a link between dynactin's role in initiating dynein-mediated transport of small organelles and the generation of cytoplasmic pulling forces.

Dynein accumulates at and tracks with growing MT plus ends in species ranging from fungi to mammals, but requirements for MT tip tracking differ. In the *C*. *elegans* early embryo, MT tip recruitment of dynein-dynactin shares similarity with the pathway in budding yeast (dynactin depends on dynein and LIS-1) and mammalian cells/filamentous fungi (dynein depends on dynactin). Surprisingly, similar to what was reported for the fungus *U*. *maydis* [65], accumulation of dynein-dynactin at MT tips does not require a CLIP-170-like protein in *C*. *elegans*. Instead, dynactin is likely directly recruited by EBP-2, one of the three EB homologs. Work in mouse fibroblasts knocked out for tubulin tyrosine ligase showed that decreased tyrosinated tubulin levels displaced CLIP-170 and p150 from MT tips [25]. By contrast, we show that MT tip targeting of *C*. *elegans* dynactin is independent of tubulin tyrosination, possibly because there is no requirement for a CLIP-170 homolog. Overall, our analysis of dynein-dynactin targeting to MT tips in *C*. *elegans* highlights the evolutionary plasticity of +TIP networks.

Our characterization of engineered *p150*^*dnc-1*^ mutants establishes the functional hierarchy among p150^DNC-1^'s tandem arrangement of MT binding regions: the CAP-Gly domain clearly provides the main activity, while the adjacent basic region plays an auxiliary role. Together with previous work in human cells [43], our results support the idea that alternative splicing of p150's basic region constitutes a conserved mechanism in animals for fine-tuning dynactin's affinity for MTs.

In budding yeast, dynein must first be targeted to MT tips prior to associating with cortical anchors [35,67]. Our results indicate that this pathway may not be used in *C*. *elegans*, as cortical accumulation of dynein-dynactin in the early embryo was unaffected in the *p150*^*dnc-1*^*(G45R)* mutant and after depletion of EBP-2, which displaced the majority of dynactin and dynein from MT tips. In agreement with normal cortical targeting of the motor, dynein-dependent cortical pulling forces remained robust in *p150*^*dnc-1*^ CAP-Gly mutants, although defects in spindle rocking indicate that the p150^DNC-1^ CAP-Gly domain does contribute to proper cortical force generation in anaphase. Importantly, depletion of EBP-2 even appeared to enhance cortical pulling during posterior spindle displacement. Thus, our results argue that dynein is recruited by its cortical anchors directly from the cytoplasm, and that dynein-dependent cortical pulling is therefore mechanistically uncoupled from prior MT tip tracking of the motor (Fig 6D). Surprisingly, even the *p150*^*dnc-1*^*(G45R +* Δ*exon 4–5)* mutant, which shows the most severe reduction in dynactin levels at MT tips (15 ± 4% of controls), is viable and fertile, suggesting that MT tip tracking of dynein-dynactin is by and large dispensable for development.

If not delivery of dynein to the cell cortex via MTs, what is the purpose of dynactin's MT binding activity? We found that *p150*^*dnc-1*^ CAP-Gly mutants have defects in the centration and rotation of the NCC, which consists of the two centrosomes and the associated female and male pronucleus. Experimental work and biophysical modelling support the idea that centration forces in the one-cell embryo are generated by dynein-mediated cytoplasmic pulling [5,63,64], although a centration/rotation model based on cortical pulling has also been proposed [68]. In the cytoplasmic pulling model, dynein works against viscous drag as it transports small organelles (e.g. endosomes, lysosomes, yolk granules) along MTs towards centrosomes, which generates pulling forces on MTs that move the NCC. Prior work showed that movements of early endosomes and centrosomes are correlated, and RNAi-mediated depletion of adaptor proteins that tether dynein to early endosomes and lysosomes inhibited centration, indicating that there is a functional link between organelle transport and cytoplasmic pulling forces [5]. In agreement with this idea, the *p150*^*dnc-1*^*(G45R +* Δ*exon 4–5)* mutant not only inhibited centration but also significantly decreased the number of early endosomes that displayed directed movement toward centrosomes. This effect on early endosome transport is consistent with the p150 CAP-Gly domain's role in initiating dynein-mediated transport, which is well-established in the context of retrograde axonal transport in neurons [30–32]. Compromising the efficiency with which organelle transport is initiated is predicted to decrease cytoplasmic pulling forces, because the magnitude of the net pulling force acting on centrosomes is proportional to the number of organelles travelling along MTs.

The frequency of centrosome-directed early endosome movement was also decreased in the *tba-1/2(YA/YA)* mutant, which severely reduced the levels of tubulin tyrosination in the early embryo. This fits well with recent work *in vitro* demonstrating that the interaction between the p150 CAP-Gly domain and tyrosinated MTs enhances the efficiency with which processive motility of dynein-dynactin is initiated [26]. Furthermore, a recent study in neurons provided evidence that initiation of retrograde transport in the distal axon is regulated by tubulin tyrosination [69]. Interestingly, depletion of EBP-2, both on its own and in the *tba-1/2(YA/YA)* mutant, also decreased early endosome transport. This suggests that EBP-2 promotes dynein-mediated transport initiation from MT tips, presumably through its interaction with the p150^DNC-1^ CAP-Gly domain, and that it can do so even in the absence of tyrosinated tubulin. In agreement with this idea, we observed that dynactin was still recruited to MT tips in the tubulin tyrosine mutant. Importantly, in addition to lowering the frequency of early endosome transport, the *tba-1/2(YA/YA)* mutant also affected centration of the NCC, and depletion of EBP-2 in the *tba-1/2(YA/YA)* mutant exacerbated the centration defect, as predicted by the centrosome-organelle mutual pulling model (Fig 6D).

Why do the *p150*^*dnc-1*^ CAP-Gly and tubulin tyrosine mutants affect centration of the NCC, but not pronuclear migration until pronuclear meeting? One plausible explanation is that during pronuclear migration the male and female pronuclei, which are large (~10 μm diameter) and equal in size, assist each other's movement as dyneins anchored on the female pronucleus walk along MTs nucleated by the centrosomes attached to the male pronucleus [70]. By contrast, during centration, the two pronuclei must be moved in the same direction, which might render cytoplasmic pulling forces more sensitive to changes in centrosome-directed transport of small (~1 μm diameter) organelles.

Finally, our data suggest that MT binding by dynactin contributes to chromosome congression. The effect is unlikely an indirect consequence of the delay in spindle orientation along the A-P axis, as chromosome congression problems were not observed after *gpr-1/2(RNAi)*, which also causes spindle orientation defects. Likewise, normal chromosome congression after *ebp-2(RNAi)* suggests that the defect in *p150*^*dnc-1*^ CAP-Gly mutants is not due to delocalization of dynactin from MT tips. Therefore, it is likely that the contribution to chromosome congression comes from the p150^DNC-1^ CAP-Gly domain pool at kinetochores, where it could aid in the capture of MTs. The decrease in embryonic viability in *p150*^*dnc-1*^ CAP-Gly mutants after inhibition of the SAC indicates that the chromosome congression defects persist in later embryonic divisions.

In summary, our work demonstrates that dynactin's MT binding activity is functionally relevant in the context of embryonic cell division. Unlike previous work that addressed p150 CAP-Gly domain function in *D*. *melanogaster* S2 cells [33], we do not observe defects in bipolar spindle formation in *p150*^*dnc-1*^ CAP-Gly mutants. Instead, the most striking consequence of inhibiting p150^DNC-1^ CAP-Gly function or tubulin tyrosination is defective centrosome centration, which we propose is a consequence of defective initiation of dynein-mediated organelle transport, in agreement with the centrosome-organelle mutual pulling model [5]. The transport initiation function of p150's CAP-Gly domain is likely generally relevant in circumstances where positioning of subcellular structures depends on dynein-mediated cytoplasmic pulling, for example the centration of sperm asters in the large eggs of amphibians and sea urchins [6,71–73].

## Materials and methods

### Worm strains

Worm strains used in this study are listed in S1 Table. Worms were maintained at 16, 20 or 25°C on standard NGM plates seeded with OP50 bacteria. A Mos1 transposon-based strategy (MosSCI) was used to generate strains stably expressing EBP-2::mKate2 and mKate2::EBP-1 [74]. Transgenes were cloned into pCFJ151 for insertion on chromosome II (ttTi5605 locus), and transgene integration was confirmed by PCR. The following alleles were generated by marker-free CRISPR-Cas9-based genome editing, as described previously [75,76]: *gfp*::*p50*^*dnc-2*^, *dynein heavy chain*^*dhc-1*^::*gfp*, *p150*^*dnc-1*^*(F26L)*, *p150*^*dnc-1*^*(G33S)*, *p150*^*dnc-1*^*(G45R)*, *p150*^*dnc-1*^*(exon 4-5-6 fusion)*, *p150*^*dnc-1*^*(*Δ*exon 4 + exon 5–6 fusion)*, *p150*^*dnc-1*^*(*Δ*exon 5 + exon 3–4 fusion)*, *p150*^*dnc-1*^*(*Δ*exon 4–5)*, *p150*^*dnc-1*^ *null*, α*-tubulin*^*tba-1*^*(Y454A)*, α*-tubulin*^*tba-2*^*(Y448A)*, *CLIP-170*^*clip-1*^ *null*, and *p150*^*dnc-1*^::*3xflag*. Genomic sequences targeted by sgRNAs are listed in S2 Table. The modifications were confirmed by sequencing and strains were outcrossed 6 times with the wild-type N2 strain. Other fluorescent markers were subsequently introduced by mating. The *p150*^*dnc-1*^*(G33S)* allele and the *p150*^*dnc-1*^ *null* allele were maintained using the GFP-marked genetic balancer nT1 [qIs51]. Homozygous F1 progeny from balanced heterozygous mothers were identified by the absence of GFP fluorescence. None of the homozygous F1 *p150*^*dnc-1*^ *null* progeny reached adulthood, and homozygous F2 *p150*^*dnc-1*^*(G33S)* progeny died during embryogenesis.

### RNA interference

For production of double-stranded RNA (dsRNA), oligos with tails containing T3 and T7 promoters were used to amplify regions from N2 genomic DNA or cDNA. Primers used for dsRNA production are listed in S3 Table. PCR reactions were cleaned (NucleoSpin Clean-up, Macherey-Nagel) and used as templates for T3 and T7 transcription reactions (MEGAscript, Invitrogen). Transcription reactions were cleaned (NucleoSpin RNA Clean-up, Macherey-Nagel) and complementary single-stranded RNAs were annealed in soaking buffer (3x soaking buffer is 32.7 mM Na_2_HPO_4_, 16.5 mM KH_2_PO_4_, 6.3 mM NaCl, 14.1 mM NH_4_Cl). dsRNAs were delivered by injecting L4 hermaphrodites, and animals were processed for live imaging after incubation at 20°C for 24 h or 48 h for partial and penetrant depletions, respectively.

### Antibodies

An affinity-purified rabbit polyclonal antibody against the N-terminal region of dynein intermediate chain^DYCI-1^ (residues 1–177) was generated as described previously [77]. In brief, a GST fusion was expressed in *E*. *coli*, purified, and injected into rabbits. Serum was affinity purified on a HiTrap *N*-hydroxysuccinimide column (GE Healthcare) against covalently coupled DYCI-1_1−177_. Antibodies against p150^DNC-1^ (GC2) and p50^DNC-2^ (GC5) were described previously [78].

### Indirect immunofluorescence

For immunofluorescence of *C*. *elegans* embryos, 10–12 adult worms were dissected into 3 μL of M9 buffer (86 mM NaCl, 42 mM Na_2_HPO_4_, 22 mM KH_2_PO_4_, 1 mM MgSO_4_) on a poly-*L*-lysine-coated slide. A 13 mm^2^ round coverslip was placed on the 3 μl drop, and slides were plunged into liquid nitrogen. After rapid removal of the coverslip ("freeze-cracking"), embryos were fixed in −20°C methanol for 20 min. Embryos were re-hydrated for 2 x 5 min in PBS (137 mM NaCl, 2.7 mM KCl, 8.1 mM Na_2_HPO_4_, and 1.47 mM KH_2_PO_4_), blocked with AbDil (PBS with 2% BSA, 0.1% Triton X-100) in a humid chamber at room temperature for 30 minutes, and incubated with primary antibodies [mouse monoclonal anti-α-tubulin DM1A (1:1000) and rat monoclonal anti-tyrosinated α-tubulin YL1/2 (1:500)] for 2 h at room temperature. After washing for 4 x 5 min in PBS, embryos were incubated with secondary antibodies conjugated with fluorescent dyes [Alexa Fluor 488 goat anti-rat IgG (1:1000) and Alexa Fluor 568 goat anti-mouse IgG (1:1000); Life Technologies—Molecular Probes] for 1h at room temperature. Embryos were washed for 4 x 5 min in PBS and mounted in Prolong Gold with DAPI stain (Invitrogen).

Images were recorded on an inverted Zeiss Axio Observer microscope at 1 x 1 binning with a 100x NA 1.46 Plan-Apochromat objective and an Orca Flash 4.0 camera (Hamamatsu). Image files were imported into Fiji for further processing.

### Immunoblotting

For each condition, 100 worms were collected into 1 mL M9 buffer and washed 3 x with M9 buffer and once with M9 / 0.05% Triton X-100. To 100 μL of worm suspension, 33 μL 4x SDS-PAGE sample buffer [250 mM Tris-HCl, pH 6.8, 30% (v/v) glycerol, 8% (w/v) SDS, 200 mM DTT and 0.04% (w/v) bromophenol blue] and ~20 μL of glass beads were added. Samples were incubated for 3 min at 95°C and vortexed for 2 x 5 min. After centrifugation at 20000 x g for 1 min at room temperature, supernatants were collected. Proteins were resolved by 7.5% or 10% SDS-PAGE and transferred to 0.2 μm nitrocellulose membranes (Hybond ECL, Amersham Pharmacia Biotech). Membranes were rinsed 3 x with TBS (50 mM Tris-HCl, pH 7.6, 145 mM NaCl), blocked with 5% non-fat dry milk in TBST (TBS / 0.1% Tween 20) and probed at 4°C overnight with the following primary antibodies: mouse monoclonal anti-FLAG M2 (Sigma, 1:1000), mouse monoclonal anti-α-tubulin B512 (Sigma, 1:5000), rat monoclonal anti-tyrosinated α-tubulin YL1/2 (Bio-Rad Laboratories, 1:5000), rabbit polyclonal anti-DYCI-1 (GC1, 1:1000), rabbit polyclonal anti-DNC-1 (GC2, 1:1000), and rabbit polyclonal anti-DNC-2 (GC5, 1:5000). Membranes were washed 5 x with TBST, incubated with goat secondary antibodies coupled to HRP (JacksonImmunoResearch, 1:5000) for 1 hour at room temperature, and washed again 3 x with TBST. Proteins were detected by chemiluminescence using Pierce ECL Western Blotting Substrate (Thermo Scientific) and X-ray film (Fuji).

### Reverse transcription PCR

Total RNA was isolated from adult hermaphrodites using the TRIzol Plus RNA Purification Kit (Invitrogen). After 3 washes with M9, pelleted worms were homogenized in 200 μL of TRIzol reagent with a pellet pestle homogenizer and incubated at room temperature for 5 min. After addition of 40 μL chloroform, samples were shaken vigorously by hand, incubated at room temperature for 3 min, and centrifuged at 12000 x g for 15 min at 4°C. The upper phase containing the RNA was transferred to an RNase-free tube and an equal volume of 70% ethanol was added. Further RNA purification steps were performed according to the manufacturer's instructions. Purified RNA was treated with DNase I (Thermo Scientific), and cDNA was synthesized with the iScript Select cDNA Synthesis Kit (Bio-Rad Laboratories). The following oligos were used for the PCR reactions in S3B Fig: forward oligo on *p150*^*dnc-1*^ exon 3 (GAATGTCACCTGCTGCTT); forward oligo on *p150*^*dnc-1*^ exon 4 (AAAGCGGTCTACAACTCC); reverse oligo on *p150*^*dnc-1*^ exon 5 (GATTGCGATAAGTTGGAGA); reverse oligo on *p150*^*dnc-1*^ exon 6 (AGTAGTCGTGGACGCTTT). For the SL1 PCR shown in S3D Fig, the following oligos were used: forward oligo on SL1 (GGTTTAATTACCCAAGTTTGA); reverse oligo on *p150*^*dnc-1*^ exon 6 (TCCAGTATCATCAATCTTCTT).

### Embryonic viability

Embryonic viability tests were performed at 20°C. L4 hermaphrodites were grown on NGM plates with OP50 bacteria for 40 h at 20°C, then singled-out to mating plates (NGM plates with a small amount of OP50 bacteria). After 8 h, mothers were removed and the number of hatched and unhatched embryos on each plate was determined 16 h later.

### Live imaging of embryos

Gravid hermaphrodite worms were dissected in a watch glass filled with Egg Salts medium (118mM KCl, 3.4 mM MgCl_2_, 3.4 mM CaCl_2_, 5 mM HEPES, pH 7.4), and embryos were mounted onto a fresh 2% agarose pad. Imaging was performed in rooms kept at 20°C. Embryos co-expressing GFP::histone H2B and GFP::γ-tubulin were imaged on an Axio Observer microscope (Zeiss) equipped with an Orca Flash 4.0 camera (Hamamatsu), a Colibri.2 light source, and controlled by ZEN software (Zeiss). Embryos expressing GFP::p50^DNC-2^, dynein heavy chain^DHC-1^::GFP, EBP-2::mKate2, and mCherry::RAB-5 were imaged on a Nikon Eclipse Ti microscope coupled to an Andor Revolution XD spinning disk confocal system composed of an iXon Ultra 897 CCD camera (Andor Technology), a solid-state laser combiner (ALC-UVP 350i, Andor Technology), and a CSU-X1 confocal scanner (Yokogawa Electric Corporation), controlled by Andor IQ3 software (Andor Technology).

### Imaging conditions and image analysis

All imaging was performed in one-cell embryos unless otherwise indicated. Image analysis was performed using Fiji software (Image J version 2.0.0-rc-56/1.51h).

#### Pronuclear migration, centrosome positioning, centrosome-centrosome distance, and orientation of centrosome-centrosome axis

Time-lapse sequences of GFP::histone H2B and GFP::γ-tubulin, consisting of 7 x 1 μm z-stacks for GFP fluorescence and one central differential interference contrast (DIC) image captured every 10 s, were recorded at 2 x 2 binning with a 63x oil immersion objective from the start of pronuclear migration until the onset of cytokinesis. Embryo length was defined as the distance between the outermost points of the egg shell visible in DIC. After maximum intensity projection of GFP z-stacks, the x and y coordinates of pronuclei and centrosomes were recorded over time using the MTrackJ plugin by manually clicking in the center of the centrosome or nucleus. The position of centrosomes and pronuclei along the anterior-posterior axis was then calculated relative to embryo length, with the anterior reference point set to 0%. Tracks from individual embryos were aligned relative to pronuclear meeting or NEBD.

#### Transversal oscillations of the mitotic spindle

Time-lapse sequences of GFP::γ-tubulin, consisting of 12 x 1 μm z-stacks captured every 2 s, were recorded at 2 x 2 binning with a 63x oil immersion objective from the beginning of metaphase until the end of anaphase. After maximum intensity projection of z-stacks, the x and y coordinates for centrosomes were determined over time with MTrackJ and the transversal distance of each centrosome to a line bisecting the embryo along the anterior-posterior axis was calculated.

#### Levels of GFP::p50^DNC-2^ and dynein heavy chain^DHC-1^::GFP at the nuclear envelope, kinetochores, and on the mitotic spindle

Time-lapse sequences, consisting of 8 x 1 μm z-stacks captured every 10 s, were recorded at 1 x 1 binning with a 60× NA 1.4 oil immersion objective from 40–50 s prior to pronuclear meeting until the onset of cytokinesis. Nuclear envelope (NE) signal was quantified 3 frames prior to pronuclear meeting using a maximum intensity projection of the 3 z-sections representing the best in-focus images of the NE. A 2 pixel-wide line was drawn on top of the NE along its entire circumference, and a similar line was drawn next to the NE on the cytoplasmic side around the nucleus. The mean fluorescence signal of the cytoplasmic line was then subtracted from the mean fluorescence signal of the NE line.

Kinetochore signal was measured 7–8 frames before the onset of sister chromatid separation using a maximum intensity projection of the z-stack. The top 10 local maxima intensities on kinetochores were identified using the "Find Maxima" function, and the 10 values were averaged. The mean fluorescence intensity of the spindle background close to the kinetochore region was measured and subtracted from the kinetochore signal.

Mitotic spindle signal was measured 2 frames after the onset of chromosome segregation using a maximum intensity projection of the z-stack. The mean intensity in 3 separate 10 x 10 pixel squares on the spindle was determined and the three values were averaged. The mean intensity of three equivalent squares in the cytoplasm adjacent to the spindle served as background signal and was subtracted from the spindle signal.

#### Levels of GFP::p50^DNC-2^ and dynein heavy chain^DHC-1^::GFP at MT plus ends

Time-lapse sequences, consisting of a single cortical confocal section captured every 5 s, were recorded at 1 x 1 binning with a 100× NA 1.4 oil immersion objective for 1 min beginning at metaphase. 3 images that were at least 3 frames apart from each other were used for quantifications. A circle with a 5-pixel radius was drawn around individual MT plus ends (marked by EBP-2::mKate2 or mCherry::β-tubulin) and the integrated fluorescence intensity was measured in the GFP channel. The circle was then expanded by increasing the radius by 2 pixels, and the integrated intensity of this larger circle was measured. Background was defined as the difference in integrated intensities between the larger and the smaller circle. The background value was scaled in proportion to the smaller circle and then subtracted from the integrated intensity of the smaller circle to obtain a final value for the GFP signal. For presentation in graphs, all values were normalized to the mean value of the control. For quantification of the GFP::p50^DNC-2^ signal at MT plus ends after *ebp-2(RNAi)* (Fig 1D), embryos co-expressing GFP::p50^DNC-2^ and mCherry::β-tubulin were used to locate MT plus ends at the cortex.

#### Cortical residency times of GFP::p50^DNC-2^, dynein heavy chain^DHC-1^::GFP, and EBP-2::mKate2

Time-lapse sequences, consisting of a single cortical confocal section captured every 200 ms, were recorded at 1 x 1 binning with a 100× NA 1.4 oil immersion objective for 1 min beginning at metaphase. Image sequences were analyzed using the LoG Detector (estimated blob diameter 10 pixels) and the Simple LAP Tracker (linking max distance 5 pixels; gap-closing max distance 5 pixels; gap-close max frame gap 0 frames) in the TrackMate plugin. The values for track duration were considered the cortical residency times for GFP and mKate2 puncta.

#### Levels of GFP::p50^DNC-2^ and dynein heavy chain^DHC-1^::GFP at the EMS-P2 cell border

Time-lapse sequences, consisting of 8 x 1 μm z-stacks captured every 30 s, were recorded at 1 x 1 binning with 60× NA 1.4 oil immersion objective. Four-cell embryos were imaged from the beginning of nuclear envelope breakdown in AB cells until cytokinesis of the P2 cell. The signal at the EMS-P2 cell border was measured at the time of EMS spindle rotation in maximum intensity projections of the z-stacks. The top 10 local maxima intensities on the EMS-P2 cell border were identified using the "Find Maxima" function, and the 10 values were averaged. The mean fluorescence intensity of an adjacent area (20 x 20 pixels) in the EMS cytoplasm served as background and was subtracted from the EMS-P2 cell border signal.

#### Tracking of early endosomes marked with mCherry::RAB-5

Time-lapse sequences, consisting of a single confocal section captured every 400 ms, were recorded at 1 x 1 binning with a 60× NA 1.4 oil immersion objective for 6 min beginning at pronuclear meeting. Image sequences were analyzed using the LoG Detector (estimated blob diameter 6 pixels) and the Simple LAP Tracker (linking max distance 8 pixels; gap-closing max distance 16 pixels; gap-close max frame gap 2 frames) in the TrackMate plugin. All tracks whose particles showed directed movement towards centrosomes and had a track displacement of at least 0.9 μm (5 pixels) were considered.

### Statistical analysis

Values in figures and text are reported as mean ± SEM with a 95% confidence interval. Statistical analyses was performed with GraphPad Prism 7.0 software. The type of statistical analysis performed is indicated in the figure legends. Differences were considered significant at *P* values below 0.05.

## Supporting information

S1 Fig Dynein-dynactin levels and dynamics at the cell cortex. **(A)**, **(B)** Correlation plots of GFP::p50^DNC-2^ versus EBP-2::mKate2 intensity *(A)* and dynein heavy chain^DHC-1^::GFP versus EBP-2::mKate2 intensity *(B)*, measured at the cortex of metaphase one-cell embryos. Pearson correlation coefficient (r) and *P*-value indicating statistical significance are on top right. The best-fit line of a linear regression with 95% confidence bands is also shown.
**(C)**, **(D)** Residency times of EBP-2::mKate2 *(C)* and GFP::p50^DNC-2^ *(D)* puncta at the cortex of metaphase one-cell embryos. The total number *(n)* of MT plus ends scored is indicated, derived from at least 8 embryos.
(TIF)

S2 Fig Dynein targeting to MT tips depends on dynactin. **(A)** Cortical confocal section of one-cell embryos in metaphase co-expressing endogenous dynein heavy chain^DHC-1^::GFP and EBP-2::mKate2, showing that depletion of p150^DNC-1^ delocalizes dynein from MT tips. Images are maximum intensity projections over time (12 images acquired every 5 s). Scale bar, 5 μm; insets, 2 μm.
**(B)** Quantification of dynein heavy chain^DHC-1^::GFP levels at MT plus ends using fluorescence intensity measurements at the cortex. Error bars represent the SEM with a 95% confidence interval, and *n* indicates the total number of measurements from 7–8 embryos per condition. The t-test was used to determine statistical significance. ****P < 0.0001.
**(C)** Central confocal section of metaphase one-cell embryos expressing transgene-encoded mKate2::EBP-1, demonstrating the efficiency of *ebp-1/3(RNAi)*. Images are maximum intensity projections over time (10 images acquired every 300 ms). Note that for reasons that are not clear, mKate2::EBP-1 does not localize to MT plus ends. Scale bar, 5 μm.
**(D)** Schematic of the *clip-1* locus. Mutations introduced by CRISPR-Cas9-based genome editing to generate a null allele (Δ*clip-1*) are indicated in black font.
(TIF)

S3 Fig Engineering of mutants that restrict *p150^dnc-1^* expression to single splice isoforms. **(A)** Schematic of the *p150*^*dnc-1*^ locus with engineered modifications. By deleting and/or fusing exons, *p150*^*dnc-1*^ expression was restricted to single N-terminal splice isoforms (*full length*, Δ*exon 4*, Δ*exon 5*, or Δ*exon 4–5*), as indicated on the right. Introduction of a frameshift mutation after the *p150*^*dnc-1*^ start codon generated a null allele.
**(B)** Results of reverse transcription PCRs using RNA isolated from adult worms and primer pairs that allow detection of the four splice isoforms. Primer locations and predicted sizes of PCR products for the different isoforms are indicated. Crosses (*x*) indicate that the PCR will not amplify any product. All four N-terminal splice isoforms are detected in wild-type and *p150*^*dnc-1*^ null mutant adults, whereas only one isoform is detected in each *p150*^*dnc-1*^ isoform mutant. Asterisks next to gel bands denote unspecific PCR products. M, DNA size marker.
**(C)** Embryonic viability assay for *p150*^*dnc-1*^ isoform mutants. Error bars represent the SEM with a 95% confidence interval, and *n* indicates the number of hermaphrodite mothers whose progeny was counted (> 500 total progeny per condition). Statistical significance was determined by one-way ANOVA followed by Bonferroni's multiple comparison test. ns = not significant, *P* > 0.05.
**(D)** Result of a reverse transcription PCR using RNA isolated from adult wild-type worms with one of the primers recognizing the spliced leader sequence 1 (SL1) and the other located in exon 6 of *p150*^*dnc-1*^. In *C*. *elegans*, about 70% of mRNAs are trans-spliced to one of two 22 nucleotide spliced leaders, SL1 or SL2, which replace the 5' ends of pre-mRNAs. One major product was amplified in the SL1 PCR and identified as the Δ*exon 4* isoform by sequencing. A PCR reaction with a primer recognizing SL2 did not amplify any product. M, DNA size marker.
**(E)** Immunoblot of wild-type or *p150*^*dnc-1*^::*3xflag* adult worms with an antibody against the 3xFLAG tag, showing that there is no detectable p150^DNC-1^ isoform corresponding to human p135. α-Tubulin was used as the loading control. Molecular mass is indicated in kilodaltons.
(TIF)

S4 Fig Embryonic viability and localization of *p150^dnc-1^* CAP-Gly mutants. **(A)** Embryonic viability assay for *p150*^*dnc-1*^ CAP-Gly mutants. Error bars represent the SEM with a 95% confidence interval, and *n* indicates the number of hermaphrodite mothers whose progeny was counted (> 500 total progeny per condition). Statistical significance was determined by one-way ANOVA followed by Bonferroni's multiple comparison test. ****P < 0.0001; ns = not significant, *P* > 0.05.
**(B)** Quantification of dynactin levels at the nuclear envelope, kinetochores, and the mitotic spindle for the *p150*^*dnc-1*^ mutants *G45R* and *F26L*, using fluorescence intensity measurements of GFP::p50^DNC-2^. Circles represent measurements in individual embryos. Error bars represent the SEM with a 95% confidence interval. Statistical significance was determined by one-way ANOVA followed by Bonferroni's multiple comparison test. ****P < 0.0001; ns = not significant, *P* > 0.05.
(TIF)

S5 Fig *p150^dnc-1^* CAP-Gly mutants delocalize dynein from MT tips. **(A)** Stills from time-lapse sequences in 2-cell embryos expressing dynein heavy chain^DHC-1^::GFP, demonstrating that dynein is delocalized from MT plus ends in the *p150*^*dnc-1*^*(G45R)* mutant. Scale bar, 5 μm.
**(B)** Quantification of dynein levels at MT plus ends using fluorescence intensity measurements of dynein heavy chain^DHC-1^::GFP at the cortex of metaphase one-cell embryos. Error bars represent the SEM with a 95% confidence interval, and *n* indicates the total number of individual measurements from 6–7 embryos per condition. Statistical significance was determined by one-way ANOVA followed by Bonferroni's multiple comparison test. ****P < 0.0001.
**(C)** Stills from time-lapse sequences in one-cell embryos expressing dynein heavy chain^DHC-1^::GFP, showing that the *p150*^*dnc-1*^*(G45R)* mutant reduces dynein levels on the mitotic spindle and at centrosomes, but not at the nuclear envelope and kinetochores. Scale bar, 5 μm.
**(D)** Quantification of dynein levels at the nuclear envelope, kinetochores, and the mitotic spindle for the *p150*^*dnc-1*^ mutants *G45R* and *F26L*, using fluorescence intensity measurements for dynein heavy chain^DHC-1^::GFP in images as shown in *(C)*. Circles represent measurements in individual embryos. Error bars represent the SEM with a 95% confidence interval. Statistical significance was determined by one-way ANOVA followed by Bonferroni's multiple comparison test. ***P < 0.001; ns = not significant, *P* > 0.05.
(TIF)

S6 Fig Functional analysis of *p150^dnc-1^* isoform mutants. **(A)** Migration kinetics of the male pronucleus in one-cell embryos expressing single isoforms of p150^DNC-1^. The position of the male pronucleus, marked by GFP::histone H2B, was determined along the anterior-posterior axis in images captured every 10 s. Individual traces were normalized to embryo length, time-aligned relative to pronuclear meeting, averaged for the indicated number (n) of embryos, and plotted against time. Error bars represent the SEM with a 95% confidence interval.
**(B)** Positioning of centrosomes, marked by GFP::γ-tubulin, measured in time-lapse sequences as described for *(A)* and plotted relative to nuclear envelope breakdown. Solid lines indicate the midpoint between the two centrosomes (spindle position). Anaphase begins at 200 s. Error bars represent the SEM with a 95% confidence interval.
**(C)** Angle between the centrosome-centrosome (C-C) axis and the anterior-posterior (A-P) axis in one-cell embryos at nuclear envelope breakdown (NEBD) and anaphase onset. Circles correspond to measurements in individual embryos. Error bars represent the SEM with a 95% confidence interval. Statistical significance was determined by one-way ANOVA followed by Bonferroni's multiple comparison test. **P < 0.01; ns = not significant, *P* > 0.05.
(TIF)

S7 Fig *p150^dnc-1^* CAP-Gly mutants delay chromosome congression. **(A)** Selected frames from time-lapse sequences in one-cell embryos co-expressing GFP::histone H2B and GFP::γ-tubulin, showing that congression of chromosomes is delayed in the *p150*^*dnc-1*^*(F26L)* mutant. Time is relative to nuclear envelope breakdown. Scale bar, 5 μm.
**(B)** Interval duration for nuclear envelope breakdown (NEBD) to full alignment of chromosomes and full alignment of chromosomes to anaphase (onset of sister chromatid separation). Error bars represent the SEM with a 95% confidence interval, and *n* indicates the number of embryos analyzed. Statistical significance was determined by one-way ANOVA followed by Bonferroni's multiple comparison test. ***P < 0.001.
**(C)** Embryonic viability assay for *p150*^*dnc-1*^ CAP-Gly mutants with and without depletion of the spindle assembly checkpoint component Mad1^MDF-1^. Error bars represent the SEM with a 95% confidence interval, and *n* indicates the number of hermaphrodite mothers whose progeny was counted (> 500 total progeny per condition). Statistical significance was determined by two-way ANOVA followed by Bonferroni's multiple comparison test. ***P < 0.001; *P < 0.05; ^####^*P* < 0.0001; ^###^*P* < 0.001.
(TIF)

S8 Fig Defects in the *p150^dnc-1^(G45R)* mutant are not exacerbated by the *tba-1/2(YA/YA)* mutant. **(A)** Migration kinetics of the male pronucleus, marked by GFP::histone H2B, along the anterior-posterior axis. Nuclear position was determined in images captured every 10 s, individual traces were normalized to embryo length, time-aligned relative to pronuclear meeting, averaged for the indicated number (n) of embryos, and plotted against time. Error bars represent the SEM with a 95% confidence interval.
**(B)** Angle between the centrosome-centrosome (C-C) axis and the anterior-posterior (A-P) axis in one-cell embryos at nuclear envelope breakdown (NEBD) and anaphase onset. Circles correspond to measurements in individual embryos. Error bars represent the SEM with a 95% confidence interval. The t-test was used to determine statistical significance. ns = not significant, *P* > 0.05.
(TIF)

S1 Movie Microtubule tip tracking of dynactin in the *C. elegans* early embryo. One-cell embryo in metaphase co-expressing GFP::p50^DNC-2^ and EBP-2::mKate2. The anterior side is to the left. A single confocal section in the embryo center was acquired every 0.4 s. Playback speed is 30 frames per second. Scale bar, 10 μm.
(AVI)

S2 Movie Microtubule tip tracking of dynein in the *C. elegans* early embryo. Two-cell embryo expressing dynein heavy chain^DHC-1^::GFP. The anterior side is to the left. A single confocal section near the cortex was acquired every 0.2 s. Playback speed is 30 frames per second. Scale bar, 10 μm.
(AVI)

S3 Movie The *p150^dnc-1^* CAP-Gly mutants *F26L* and *G45R* delocalize dynactin from microtubule tips—center view. One-cell embryos in metaphase expressing GFP::p50^DNC-2^. The anterior side is to the left. A single confocal section in the embryo center was acquired every 0.2 s. Playback speed is 30 frames per second. Scale bar, 10 μm.
(AVI)

S4 Movie The *p150^dnc-1^* CAP-Gly mutant *F26L* delocalizes dynactin from microtubule tips—cortical view. One-cell embryos in metaphase expressing GFP::p50^DNC-2^. The anterior side is to the left. A single confocal section at the cortex was acquired every 0.2 s. Playback speed is 30 frames per second. Scale bar, 10 μm.
(AVI)

S5 Movie The *p150^dnc-1^* CAP-Gly mutant *G45R* delocalizes dynactin from microtubule tips—cortical view. One-cell embryos in metaphase expressing GFP::p50^DNC-2^. The anterior side of the embryo is to the left. A single confocal section at the cortex was acquired every 0.2 s. Playback speed is 30 frames per second. Scale bar, 10 μm.
(AVI)

S6 Movie Centration/rotation defects in *p150^dnc-1^* CAP-Gly mutants. One-cell embryos expressing GFP-labelled histone H2B and γ-tubulin to mark chromosomes and centrosomes, respectively. The anterior side is to the left. Time lapse is 10 s and playback speed is 6 frames per second. Scale bar, 10 μm.
(AVI)

S7 Movie Delayed chromosome congression and dampened anaphase spindle oscillations in *p150^dnc-1^* CAP-Gly mutants. One-cell embryos expressing GFP-labelled histone H2B and γ-tubulin to mark chromosomes and centrosomes, respectively. The anterior side is to the left. Time lapse is 2 s and playback speed is 12 frames per second. Scale bar, 10 μm.
(AVI)

S8 Movie Centration/rotation defects in α-tubulin tyrosine mutants. One-cell embryos expressing GFP-labelled histone H2B and γ-tubulin to mark chromosomes and centrosomes, respectively. The anterior side is to the left. Time lapse is 10 s and playback speed is 6 frames per second. Scale bar, 10 μm.
(AVI)

S9 Movie Defective transport of early endosomes in the *p150^dnc-1^(G45R +* Δ*exon 4–5)* mutant. One-cell embryos during the centration phase. Early endosomes are marked by mCherry::RAB-5. The anterior side is to the left. Time lapse is 0.4 s and playback speed is 30 frames per second. Scale bar, 10 μm.
(AVI)

S1 Table Worm strains used in this study. (DOCX)

S2 Table Genomic sequences targeted by sgRNAs for CRISPR-Cas9-assisted genome editing. (DOCX)

S3 Table Oligos for double-stranded RNA production. (DOCX)
