# Supplementary figures and images for: Dynactin binding to tyrosinated microtubules promotes centrosome centration in C. elegans by enhancing dynein-mediated organelle transport
Source: PLoS Genet. 2017 Jul 31;13(7):e1006941. doi: 10.1371/journal.pgen.1006941 (PMC5552355; doi:10.1371/journal.pgen.1006941)

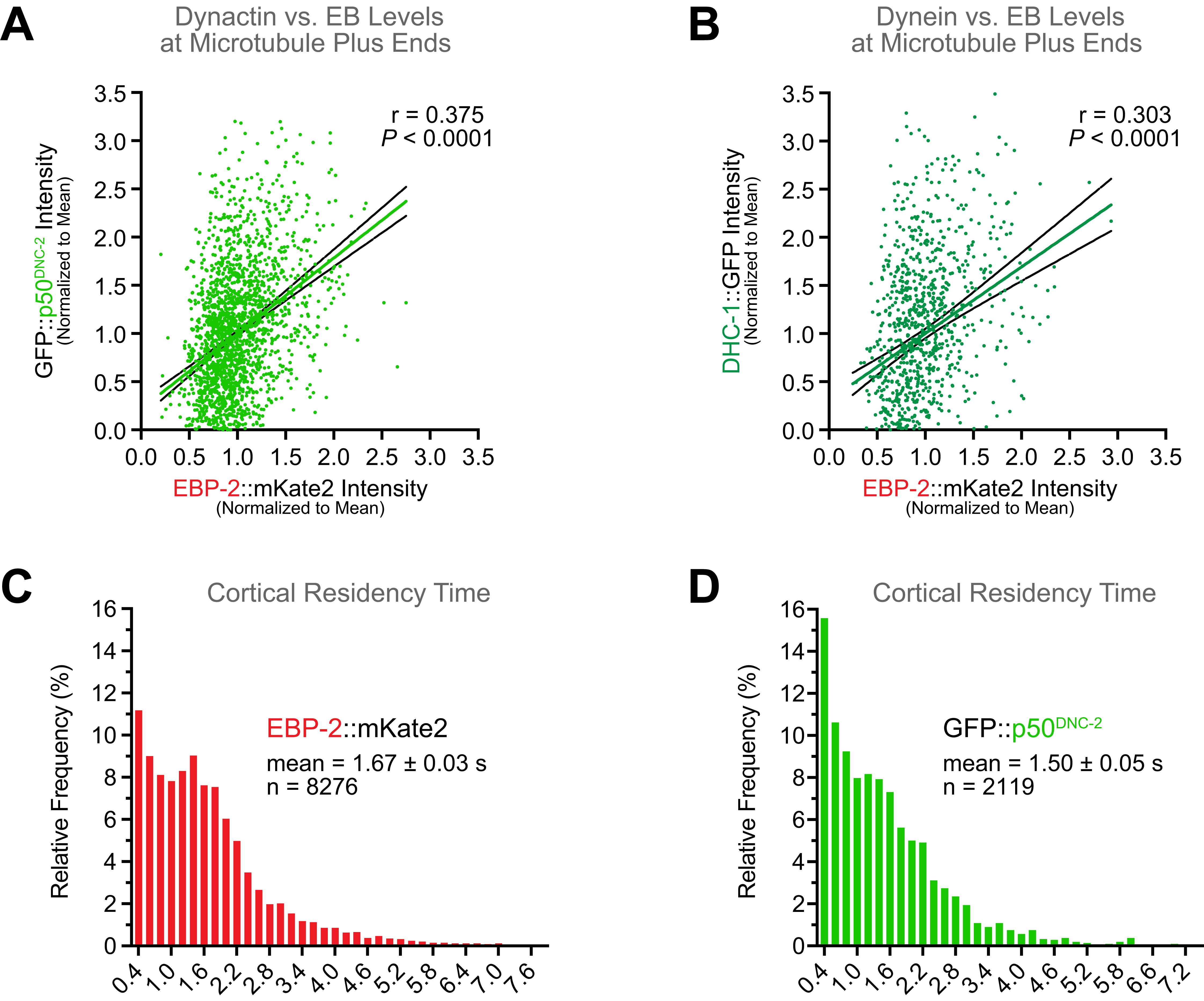

Supplement: S1 Fig — (A), (B) Correlation plots of GFP::p50DNC-2 versus EBP-2::mKate2 intensity (A) and dynein heavy chainDHC-1::GFP versus EBP-2::mKate2 intensity (B), measured at the cortex of metaphase one-cell embryos. Pearson correlation coefficient (r) and P-value indicating statistical significance are on top right. The best-fit line of a linear regression with 95% confidence bands is also shown. (C), (D) Residency times of EBP-2::mKate2 (C) and GFP::p50DNC-2 (D) puncta at the cortex of metaphase one-cell embryos. The total number (n) of MT plus ends scored is indicated, derived from at least 8 embryos. (TIF) [file pgen.1006941.s001.tif]

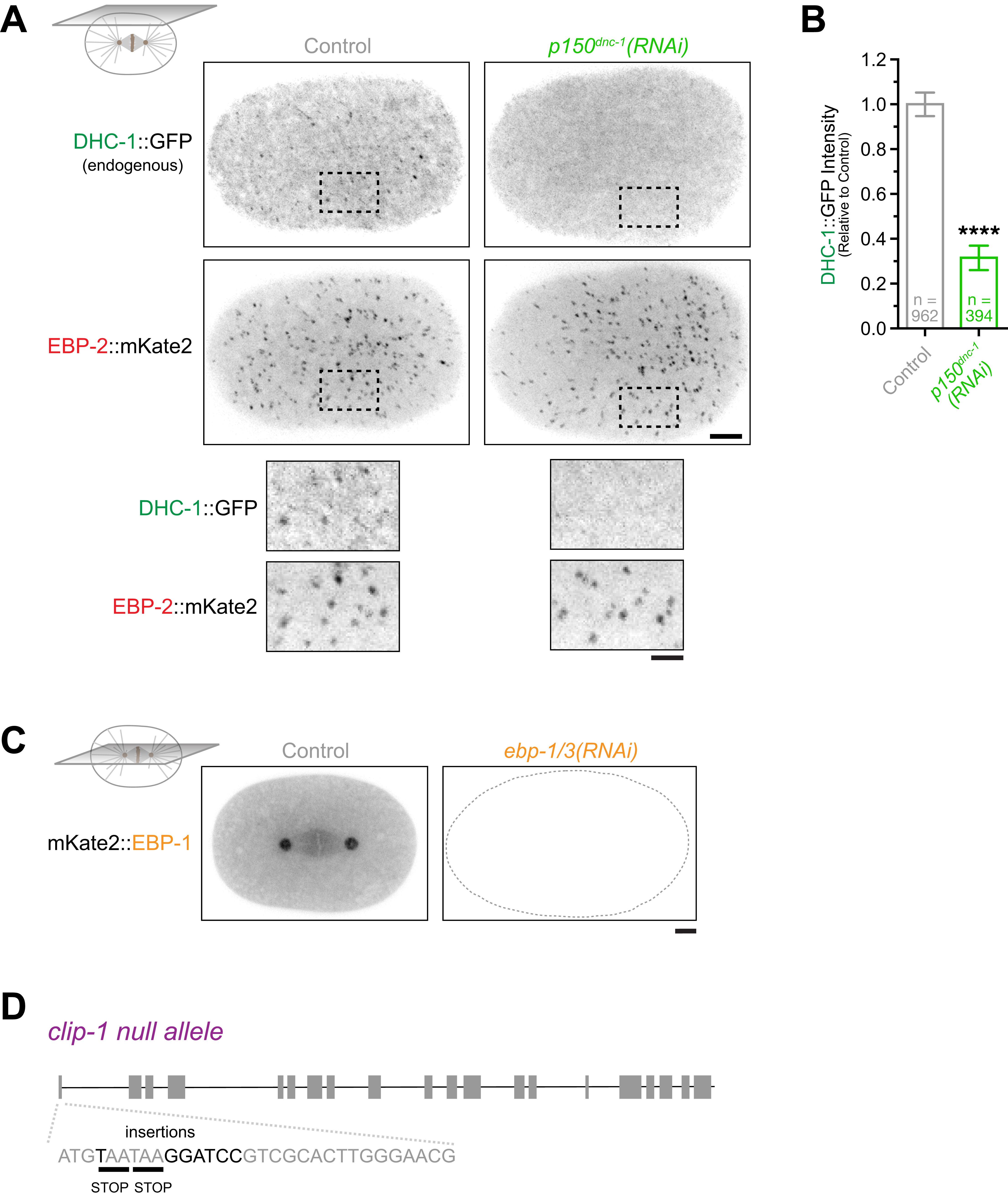

Supplement: S2 Fig — (A) Cortical confocal section of one-cell embryos in metaphase co-expressing endogenous dynein heavy chainDHC-1::GFP and EBP-2::mKate2, showing that depletion of p150DNC-1 delocalizes dynein from MT tips. Images are maximum intensity projections over time (12 images acquired every 5 s). Scale bar, 5 μm; insets, 2 μm. (B) Quantification of dynein heavy chainDHC-1::GFP levels at MT plus ends using fluorescence intensity measurements at the cortex. Error bars represent the SEM with a 95% confidence interval, and n indicates the total number of measurements from 7–8 embryos per condition. The t-test was used to determine statistical significance. ****P < 0.0001. (C) Central confocal section of metaphase one-cell embryos expressing transgene-encoded mKate2::EBP-1, demonstrating the efficiency of ebp-1/3(RNAi). Images are maximum intensity projections over time (10 images acquired every 300 ms). Note that for reasons that are not clear, mKate2::EBP-1 does not localize to MT plus ends. Scale bar, 5 μm. (D) Schematic of the clip-1 locus. Mutations introduced by CRISPR-Cas9-based genome editing to generate a null allele (Δclip-1) are indicated in black font. (TIF) [file pgen.1006941.s002.tif]

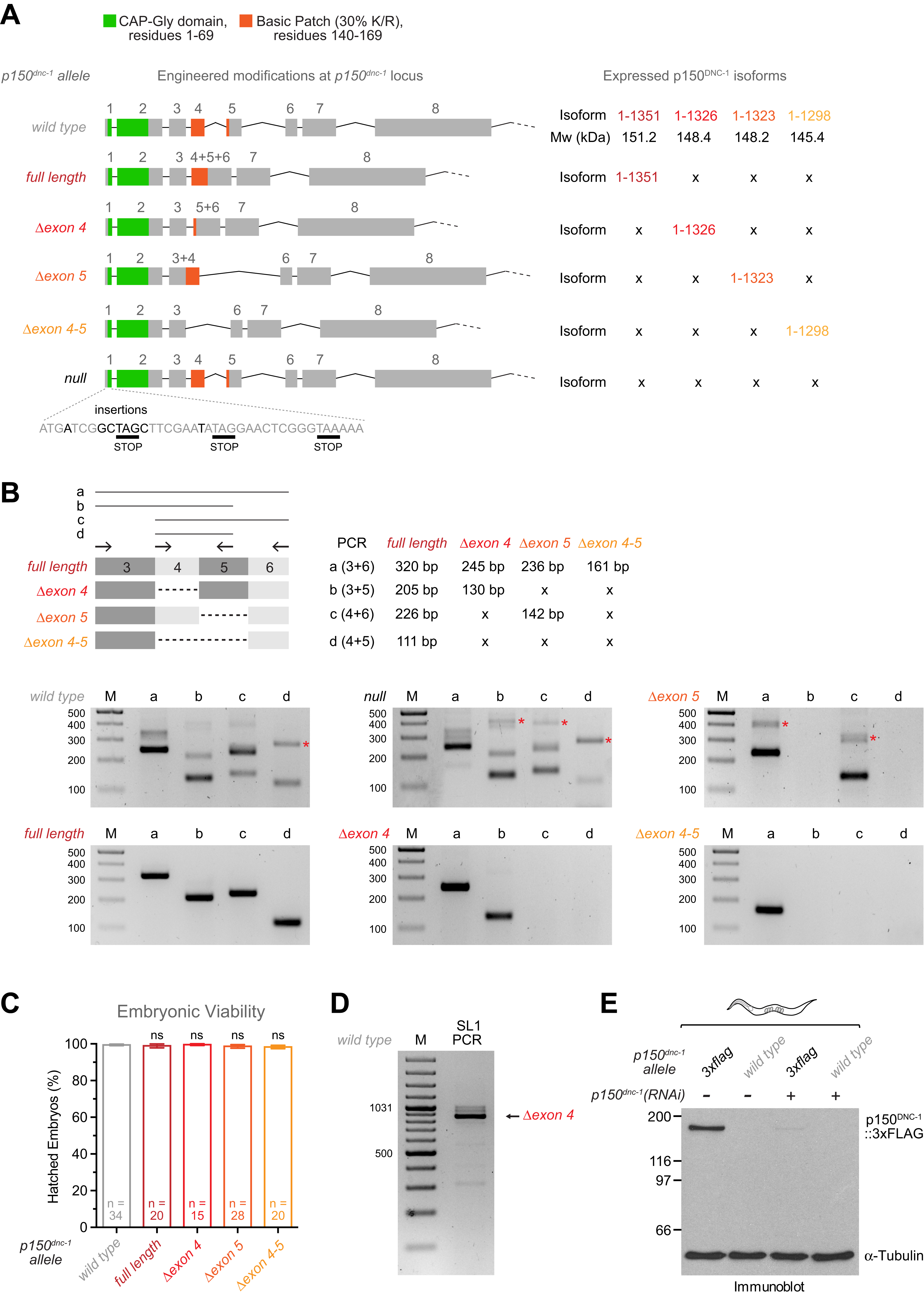

Supplement: S3 Fig — (A) Schematic of the p150dnc-1 locus with engineered modifications. By deleting and/or fusing exons, p150dnc-1 expression was restricted to single N-terminal splice isoforms (full length, Δexon 4, Δexon 5, or Δexon 4–5), as indicated on the right. Introduction of a frameshift mutation after the p150dnc-1 start codon generated a null allele. (B) Results of reverse transcription PCRs using RNA isolated from adult worms and primer pairs that allow detection of the four splice isoforms. Primer locations and predicted sizes of PCR products for the different isoforms are indicated. Crosses (x) indicate that the PCR will not amplify any product. All four N-terminal splice isoforms are detected in wild-type and p150dnc-1 null mutant adults, whereas only one isoform is detected in each p150dnc-1 isoform mutant. Asterisks next to gel bands denote unspecific PCR products. M, DNA size marker. (C) Embryonic viability assay for p150dnc-1 isoform mutants. Error bars represent the SEM with a 95% confidence interval, and n indicates the number of hermaphrodite mothers whose progeny was counted (> 500 total progeny per condition). Statistical significance was determined by one-way ANOVA followed by Bonferroni's multiple comparison test. ns = not significant, P > 0.05. (D) Result of a reverse transcription PCR using RNA isolated from adult wild-type worms with one of the primers recognizing the spliced leader sequence 1 (SL1) and the other located in exon 6 of p150dnc-1. In C. elegans, about 70% of mRNAs are trans-spliced to one of two 22 nucleotide spliced leaders, SL1 or SL2, which replace the 5' ends of pre-mRNAs. One major product was amplified in the SL1 PCR and identified as the Δexon 4 isoform by sequencing. A PCR reaction with a primer recognizing SL2 did not amplify any product. M, DNA size marker. (E) Immunoblot of wild-type or p150dnc-1::3xflag adult worms with an antibody against the 3xFLAG tag, showing that there is no detectable p150DNC-1 isoform corresponding to human p [file pgen.1006941.s003.tif]

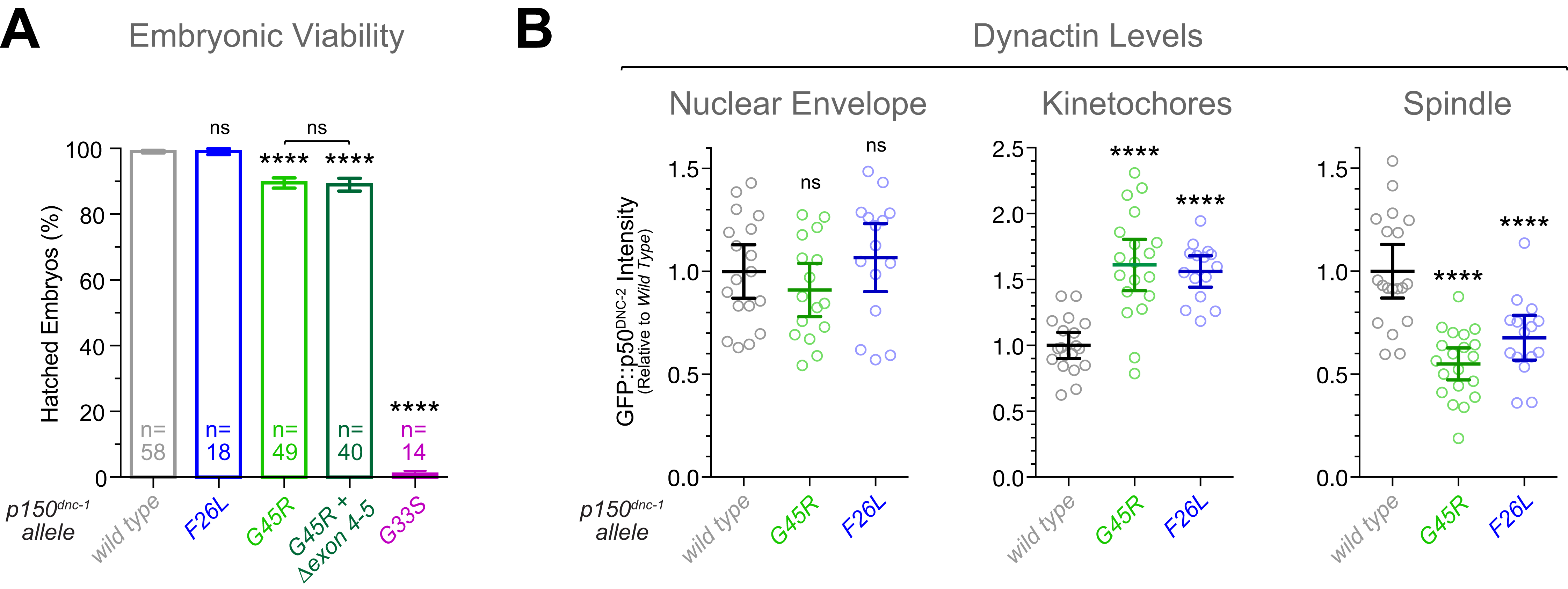

Supplement: S4 Fig — (A) Embryonic viability assay for p150dnc-1 CAP-Gly mutants. Error bars represent the SEM with a 95% confidence interval, and n indicates the number of hermaphrodite mothers whose progeny was counted (> 500 total progeny per condition). Statistical significance was determined by one-way ANOVA followed by Bonferroni's multiple comparison test. ****P < 0.0001; ns = not significant, P > 0.05. (B) Quantification of dynactin levels at the nuclear envelope, kinetochores, and the mitotic spindle for the p150dnc-1 mutants G45R and F26L, using fluorescence intensity measurements of GFP::p50DNC-2. Circles represent measurements in individual embryos. Error bars represent the SEM with a 95% confidence interval. Statistical significance was determined by one-way ANOVA followed by Bonferroni's multiple comparison test. ****P < 0.0001; ns = not significant, P > 0.05. (TIF) [file pgen.1006941.s004.tif]

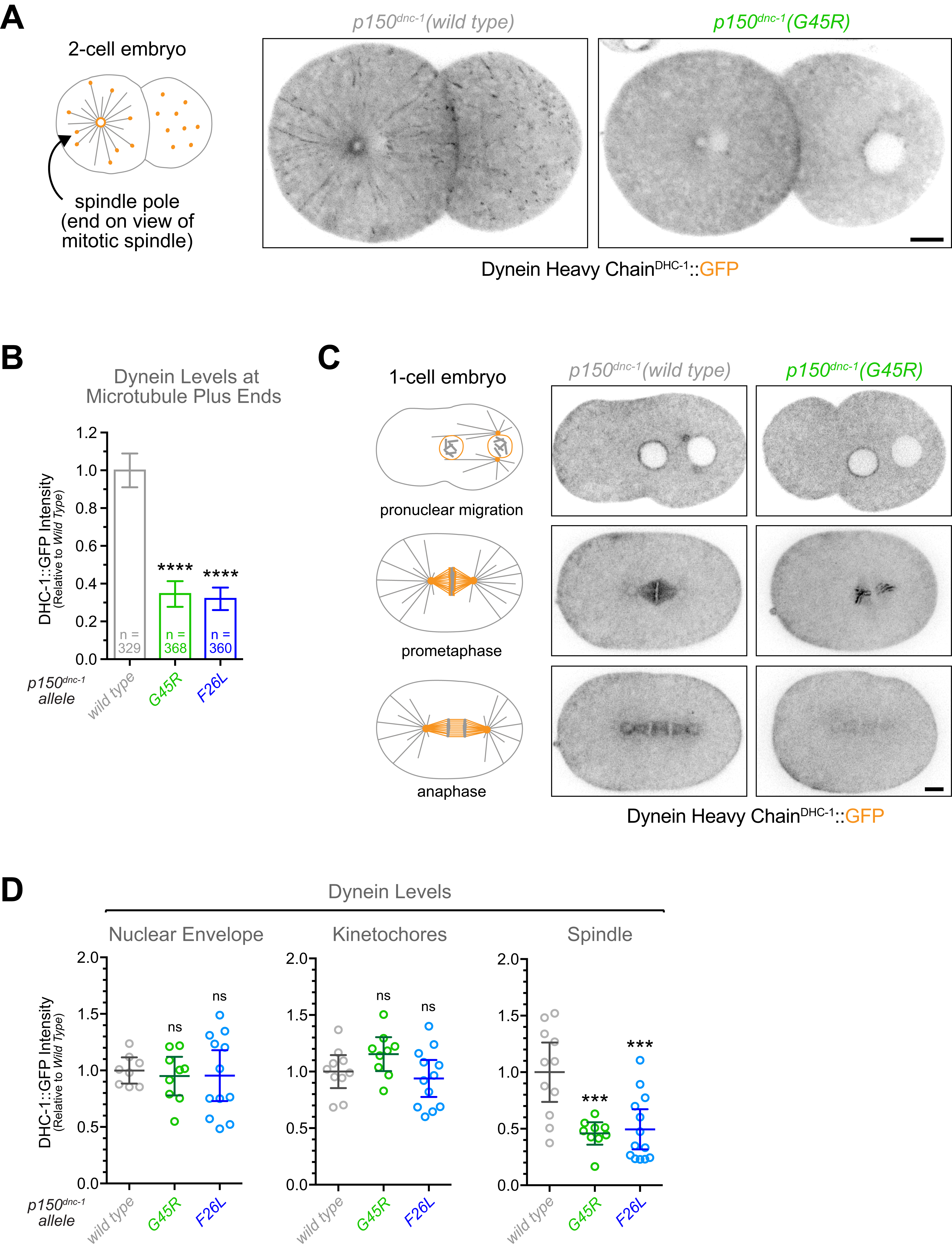

Supplement: S5 Fig — (A) Stills from time-lapse sequences in 2-cell embryos expressing dynein heavy chainDHC-1::GFP, demonstrating that dynein is delocalized from MT plus ends in the p150dnc-1(G45R) mutant. Scale bar, 5 μm. (B) Quantification of dynein levels at MT plus ends using fluorescence intensity measurements of dynein heavy chainDHC-1::GFP at the cortex of metaphase one-cell embryos. Error bars represent the SEM with a 95% confidence interval, and n indicates the total number of individual measurements from 6–7 embryos per condition. Statistical significance was determined by one-way ANOVA followed by Bonferroni's multiple comparison test. ****P < 0.0001. (C) Stills from time-lapse sequences in one-cell embryos expressing dynein heavy chainDHC-1::GFP, showing that the p150dnc-1(G45R) mutant reduces dynein levels on the mitotic spindle and at centrosomes, but not at the nuclear envelope and kinetochores. Scale bar, 5 μm. (D) Quantification of dynein levels at the nuclear envelope, kinetochores, and the mitotic spindle for the p150dnc-1 mutants G45R and F26L, using fluorescence intensity measurements for dynein heavy chainDHC-1::GFP in images as shown in (C). Circles represent measurements in individual embryos. Error bars represent the SEM with a 95% confidence interval. Statistical significance was determined by one-way ANOVA followed by Bonferroni's multiple comparison test. ***P < 0.001; ns = not significant, P > 0.05. (TIF) [file pgen.1006941.s005.tif]

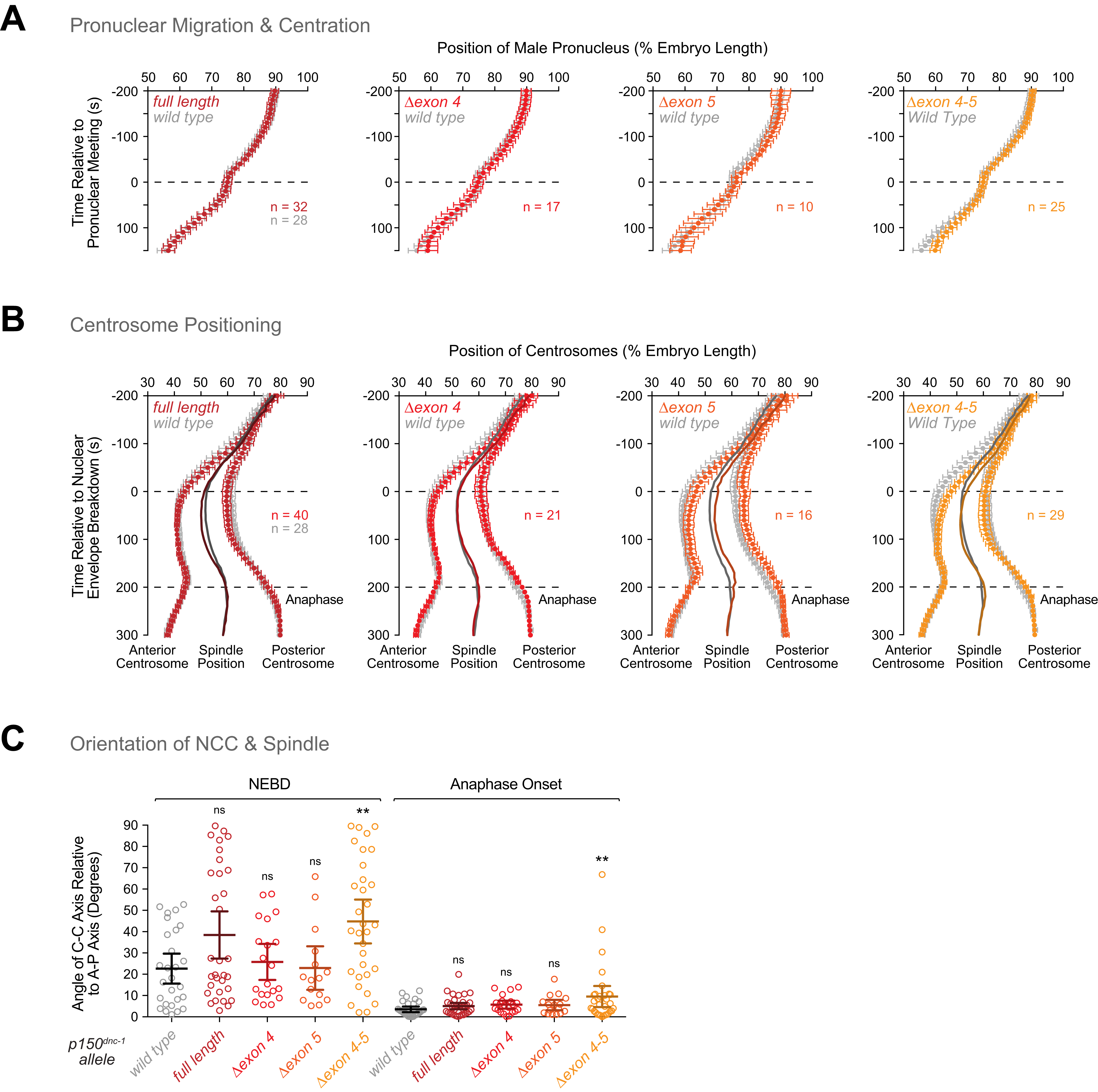

Supplement: S6 Fig — (A) Migration kinetics of the male pronucleus in one-cell embryos expressing single isoforms of p150DNC-1. The position of the male pronucleus, marked by GFP::histone H2B, was determined along the anterior-posterior axis in images captured every 10 s. Individual traces were normalized to embryo length, time-aligned relative to pronuclear meeting, averaged for the indicated number (n) of embryos, and plotted against time. Error bars represent the SEM with a 95% confidence interval. (B) Positioning of centrosomes, marked by GFP::γ-tubulin, measured in time-lapse sequences as described for (A) and plotted relative to nuclear envelope breakdown. Solid lines indicate the midpoint between the two centrosomes (spindle position). Anaphase begins at 200 s. Error bars represent the SEM with a 95% confidence interval. (C) Angle between the centrosome-centrosome (C-C) axis and the anterior-posterior (A-P) axis in one-cell embryos at nuclear envelope breakdown (NEBD) and anaphase onset. Circles correspond to measurements in individual embryos. Error bars represent the SEM with a 95% confidence interval. Statistical significance was determined by one-way ANOVA followed by Bonferroni's multiple comparison test. **P < 0.01; ns = not significant, P > 0.05. (TIF) [file pgen.1006941.s006.tif]

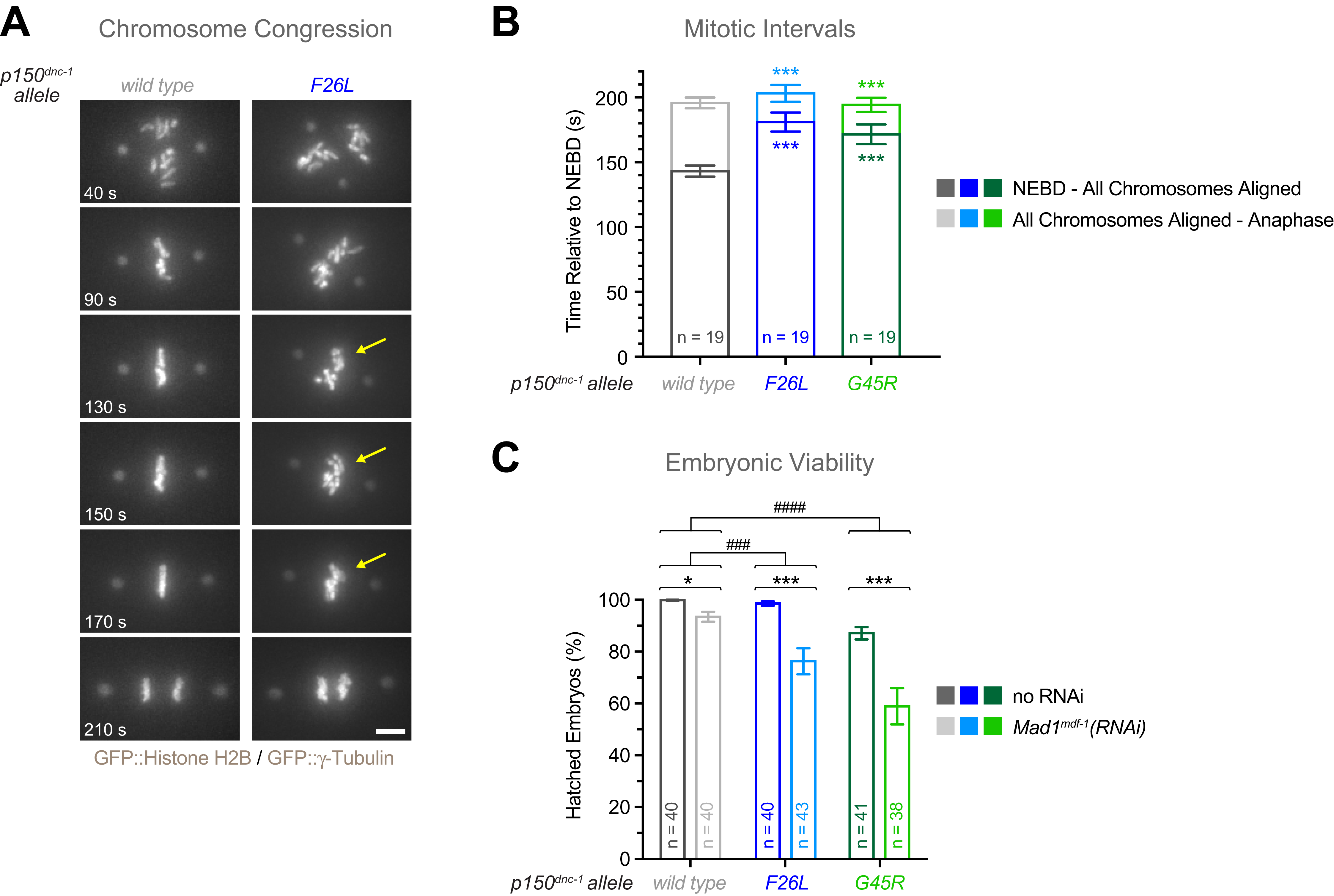

Supplement: S7 Fig — (A) Selected frames from time-lapse sequences in one-cell embryos co-expressing GFP::histone H2B and GFP::γ-tubulin, showing that congression of chromosomes is delayed in the p150dnc-1(F26L) mutant. Time is relative to nuclear envelope breakdown. Scale bar, 5 μm. (B) Interval duration for nuclear envelope breakdown (NEBD) to full alignment of chromosomes and full alignment of chromosomes to anaphase (onset of sister chromatid separation). Error bars represent the SEM with a 95% confidence interval, and n indicates the number of embryos analyzed. Statistical significance was determined by one-way ANOVA followed by Bonferroni's multiple comparison test. ***P < 0.001. (C) Embryonic viability assay for p150dnc-1 CAP-Gly mutants with and without depletion of the spindle assembly checkpoint component Mad1MDF-1. Error bars represent the SEM with a 95% confidence interval, and n indicates the number of hermaphrodite mothers whose progeny was counted (> 500 total progeny per condition). Statistical significance was determined by two-way ANOVA followed by Bonferroni's multiple comparison test. ***P < 0.001; *P < 0.05; ####P < 0.0001; ###P < 0.001. (TIF) [file pgen.1006941.s007.tif]

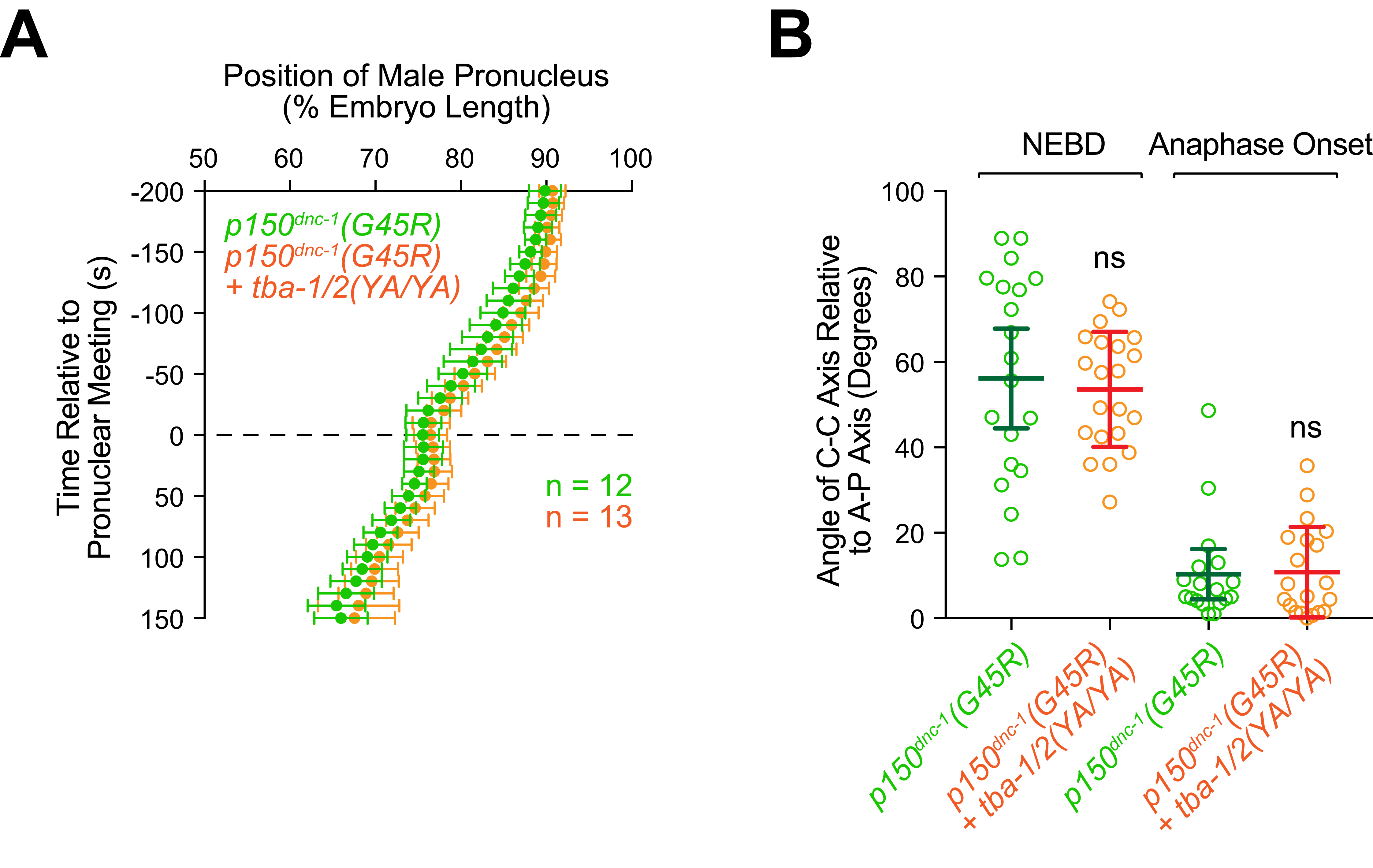

Supplement: S8 Fig — (A) Migration kinetics of the male pronucleus, marked by GFP::histone H2B, along the anterior-posterior axis. Nuclear position was determined in images captured every 10 s, individual traces were normalized to embryo length, time-aligned relative to pronuclear meeting, averaged for the indicated number (n) of embryos, and plotted against time. Error bars represent the SEM with a 95% confidence interval. (B) Angle between the centrosome-centrosome (C-C) axis and the anterior-posterior (A-P) axis in one-cell embryos at nuclear envelope breakdown (NEBD) and anaphase onset. Circles correspond to measurements in individual embryos. Error bars represent the SEM with a 95% confidence interval. The t-test was used to determine statistical significance. ns = not significant, P > 0.05. (TIF) [file pgen.1006941.s008.tif]
