# Supplementary material for: Dynactin binding to tyrosinated microtubules promotes centrosome centration in C. elegans by enhancing dynein-mediated organelle transport
Source: PLoS Genet. 2017 Jul 31;13(7):e1006941. doi: 10.1371/journal.pgen.1006941 (PMC5552355; doi:10.1371/journal.pgen.1006941)
Supplement: S1 Table — (DOCX) [file pgen.1006941.s018.docx]

**S1 Table.** **Worm strains used in this study.**

| Strain Name | Genotype |
| --- | --- |
| N2 | WT (ancestral N2 Bristol) |
| GCP181 | dnc-1[prt7(F26L)] IV |
| GCP231 | dnc-1[prt12(G33S)] IV; unc-5(e53) IV/nT1 [qIs51] (IV;V); dpy-11(e224) V/nT1 [qIs51] (IV;V) |
| GCP237 | dnc-1[prt14(Δexon 4-5)] IV |
| GCP242 | dnc-1[prt15(C-terminal 3xflag)] IV |
| GCP245 | dnc-1[prt18(G45R)] IV |
| GCP247 | dnc-1[prt8(exon 5-6 fusion)] IV; dnc-1[prt20(exon 4-5 fusion)] IV |
| GCP255 | dnc-1[prt23(knockout)]; unc-5(e53) IV/nT1 [qIs51] (IV;V); dpy-11(e224) V/nT1 [qIs51] (IV;V) |
| GCP289 | dnc-1[prt7(F26L)] IV; unc-119(ed3) III?; ruIs32[pAZ132; Ppie-1::gfp::his-58; unc-119(+)] III; ddIs6 [Ppie-1::gfp::tbg-1; unc-119(+)] V * |
| GCP290 | dnc-1[prt18(G45R)] IV; unc-119(ed3) III?; ruIs32[pAZ132; Ppie-1::gfp::his-58; unc-119(+)] III; ddIs6 [Ppie-1::gfp::tbg-1; unc-119(+)] V * |
| GCP291 | dnc-1[prt14(Δexon 4-5)] IV; unc-119(ed3) III?; ruIs32[pAZ132; Ppie-1::gfp::his-58; unc-119(+)] III; ddIs6[Ppie-1::gfp::tbg-1; unc-119(+)] V * |
| GCP292 | dnc-1[prt8(exon 5-6 fusion)] IV; dnc-1[prt20(exon 4-5 fusion)] IV; unc-119(ed3) III?; ruIs32[pAZ132; Ppie-1::gfp::his-58; unc-119(+)] III; ddIs6[Ppie-1::gfp::tbg-1; unc-119(+)] V * |
| GCP412 | dnc-1[prt7(F26L)] IV; dhc-1[C-terminal gfp] I |
| GCP413 | dnc-1[prt18(G45R)] IV; dhc-1[C-terminal gfp] I |
| GCP416 | unc-119(ed3) III; prtSi122[pRG629; Pmex-5::ebp-2::mKate2::tbb-2 3'UTR; cb-unc-119(+)] II |
| GCP417 | dnc-2[prt42(N-terminal 3xflag::gfp)] III |
| GCP430 | dnc-1[prt7(F26L)] IV; unc-119(ed3) III?; prtSi122[pRG629; Pmex-5::ebp-2::mKate2::tbb-2 3'UTR; cb-unc-119(+)] II * |
| GCP431 | dnc-1[prt18(G45R)] IV; unc-119(ed3) III?; prtSi122[pRG629; Pmex-5::ebp-2::mKate2::tbb-2 3'UTR; cb-unc-119(+)] II * |
| GCP432 | dnc-1[prt7(F26L)] IV; dnc-2[prt42(N-terminal 3xflag::gfp)] III |
| GCP433 | dnc-1[prt18(G45R)] IV; dnc-2[prt42(N-terminal 3xflag::gfp] III |
| GCP442 | tba-1[prt55(Y454A)] I |
| GCP444 | dnc-1[prt14(Δexon 4-5)] IV; dnc-2[prt42(N-terminal 3xflag::gfp)] III |
| GCP445 | dnc-1[prt8(exon 5-6 fusion)] IV; dnc-1[prt20(exon 4-5 fusion)] IV; dnc-2[prt42(N-terminal 3xflag::gfp)] III |
| GCP446 | dhc-1[C-terminal gfp] I; unc-119(ed3) III?; prtSi122[pRG629; Pmex-5::ebp-2::mKate2::tbb-2 3'UTR; cb-unc-119(+)] II * |
| GCP447 | dnc-2[prt42(N-terminal 3xflag::gfp)] III; unc-119(ed3) III?; prtSi122[pRG629; Pmex-5::ebp-2::mKate2::tbb-2 3'UTR; cb-unc-119(+)] II * |
| GCP452 | tba-2[prt57(Y448A)] I |
| GCP477 | dnc-1[prt8(exon 5-6 fusion)] IV; dnc-1[prt61(Δexon 4)] IV |
| GCP487 | tba-1[prt55(Y454A)] I; tba-2[prt58(Y448A)] I |
| GCP494 | dnc-1[prt69(exon 3-4 fusion)] IV; dnc-1[prt71(Δexon 5)] IV |
| GCP499 | dnc-1[prt8(exon 5-6 fusion)] IV; dnc-1[prt61(Δexon 4)] IV; dnc-2[prt42(N-terminal 3xflag::gfp)] III |
| GCP500 | dnc-1[prt8(exon 5-6 fusion)] IV; dnc-1[prt61(Δexon 4)] IV; unc-119(ed3) III?; ruIs32[pAZ132; Ppie-1::gfp::his-58; unc-119(+)] III; ddIs6 [Ppie-1::gfp::tbg-1; unc-119(+)] V * |
| GCP502 | tba-1[prt55(Y454A)] I; tba-2[prt58(Y448A)] I; dnc-2[prt42(N-terminal 3xflag::gfp)] III |
| GCP503 | tba-1[prt55(Y454A)] I; tba-2[prt58(Y448A)] I; unc-119(ed3) III?; ruIs32[pAZ132; Ppie-1::gfp::his-58; unc-119(+)] III; ddIs6 [Ppie-1::gfp::tbg-1; unc-119(+)] V * |
| GCP520 | dnc-1[prt69(exon 3-4 fusion)] IV; dnc-1[prt71(Δexon 5)] IV; dnc-2[prt42(N-terminal 3xflag::gfp)] III |
| GCP522 | dnc-1[prt69(exon 3-4 fusion)] IV; dnc-1[prt71(Δexon 5)] IV; unc-119(ed3) III?; ruIs32[pAZ132; Ppie-1::gfp::his-58; unc-119(+)] III; ddIs6 [Ppie-1::gfp::tbg-1; unc-119(+)] V * |
| GCP534 | dnc-1[prt7(F26L)] IV; dnc-2[prt42(N-terminal 3xflag::gfp)] III; unc-119(ed3) III?; prtSi122[pRG629; Pmex-5::ebp-2::mKate2::tbb-2 3'UTR; cb-unc-119(+)] II * |
| GCP535 | dnc-1[prt18(G45R)] IV; dnc-2[prt42(N-terminal 3xflag::gfp)] III; unc-119(ed3) III?; prtSi122[pRG629; Pmex-5::ebp-2::mKate2::tbb-2 3'UTR; cb-unc-119(+)] II * |
| GCP562 | dnc-1[prt18(G45R)] IV; dnc-1[prt74(Δexon 4-5)] IV; unc-119(ed3) III?; ruIs32[pAZ132; Ppie-1::gfp::his-58; unc-119(+)] III; ddIs6 [Ppie-1::gfp::tbg-1; unc-119(+)] V * |
| GCP575 | dnc-1[prt18(G45R)] IV; tba-1[prt55(Y454A)] I; tba-2[prt58(Y448A)] I; unc-119(ed3) III?; ruIs32[pAZ132; Ppie-1::gfp::his-58; unc-119(+)] III; ddIs6 [Ppie-1::gfp::tbg-1; unc-119(+)] V * |
| GCP581 | dnc-1[prt7(F26L)] IV; dhc-1[C-terminal gfp] I; unc-119(ed3) III?; prtSi122[pRG629; Pmex-5::ebp-2::mKate2::tbb-2 3'UTR; cb-unc-119(+)] II * |
| GCP582 | dnc-1[prt18(G45R)] IV; dhc-1[C-terminal gfp] I; unc-119(ed3) III?; prtSi122[pRG629; Pmex-5::ebp-2::mKate2::tbb-2 3'UTR; cb-unc-119(+)] II * |
| GCP585 | dnc-1[prt18(G45R)] IV; dnc-1[prt74(Δexon 4-5)] IV; dnc-2[prt42(N-terminal 3xflag::gfp)] III; unc-119(ed3) III?; prtSi122[pRG629; Pmex-5::ebp-2::mKate2::tbb-2 3'UTR; cb-unc-119(+)] II * |
| GCP586 | dnc-1[prt18(G45R)] IV; dnc-1[prt74(Δexon 4-5)] IV; unc-119(ed3) III?; ltIs79[pAA196; Ppie-1::mCherry::rab-5; unc-119(+)]; pwIs116[Prme-2::rme-2::gfp::rme-2 3'UTR; unc-119(+)] * |
| GCP587 | tba-1[prt55(Y454A)] I; tba-2[prt58(Y448A)] I; unc-119(ed3) III?; ltIs79[pAA196; Ppie-1::mCherry::rab-5; unc-119(+)]; pwIs116[Prme-2::rme-2::gfp::rme-2 3'UTR; unc-119(+)] * |
| GCP604 | clip-1[prt92(knockout)] III; dnc-2[prt42(N-terminal 3xflag::gfp)] III; unc-119(ed3) III?; prtSi122[pRG629; Pmex-5::ebp-2::mKate2::tbb-2 3'UTR; cb-unc-119(+)] II * |
| GCP606 | unc-119(ed3) III; prtSi135[pRG777; Pmex-5::mKate2::ebp-1::tbb-2 3'UTR; cb-unc-119(+)] II |
| GCP632 | dnc-2[prt42(N-terminal 3xflag::gfp)] III; unc-119(ed3) III?; weIs21 [pJA138; Ppie-1::mCherry::β-tubulin::pie-1 3’UTR)] IV * |
| OD179 | unc-119(ed3) III?; ltIs79[pAA196; Ppie-1::mCherry::rab-5; unc-119(+)]; pwIs116[Prme-2::rme-2::gfp::rme-2 3'UTR; unc-119(+)] * |
| OD2955 | dhc-1[C-terminal gfp] I |
| TH32 | unc-119(ed3) III?; ruIs32[pAZ132; Ppie-1::gfp::his-58; unc-119(+)] III; ddIs6[Ppie-1:gfp::tbg-1; unc-119(+)] V * |

* unc-119(ed3) IIII? was present in parental strains, but these strains have not been sequenced to determine whether the unc-119 gene contains the ed3 mutation.
