# Supplementary material for: Dynactin binding to tyrosinated microtubules promotes centrosome centration in C. elegans by enhancing dynein-mediated organelle transport
Source: PLoS Genet. 2017 Jul 31;13(7):e1006941. doi: 10.1371/journal.pgen.1006941 (PMC5552355; doi:10.1371/journal.pgen.1006941)
Supplement: S2 Table — (DOCX) [file pgen.1006941.s019.docx]

**S2 Table. Genomic sequences targeted by sgRNAs for CRISPR-Cas9-assisted genome editing.**

| Gene ID | Gene Name | Modification | sgRNA target site (5'-3' sequence) and PAM motif |
| --- | --- | --- | --- |
| C28H8.12 | *dnc-2* | N-terminal 3xflag::gfp | GCGTCAAAATGTCATCTATTGG |
| C47B2.3 | *tba-2* | Y448A | AACGAGGGAGGAGAAGAGGAGG |
|  |  |  | GGAGGAGAAGAGGAGGGAGAGG |
| F26E4.8 | *tba-1* | Y454A | AACGAGGGAGGAAACGAGGAGG |
|  |  |  | AGGGAGGAAACGAGGAGGAAGG |
|  |  |  | TTATGCAGATTGGAGCAGGCGG |
| M01A8.2 | *clip-1* | Premature stop codon  (null mutant) | ACAATGAATAAGTCGCACTTGG |
|  |  |  | CAATGAATAAGTCGCACTTGGG |
|  |  |  | TGCGACTTATTCATTGTATGGG |
| T14B4.7 | *dpy-10* | cn64 | GCTACCATAGGCACCACGAGCGG |
| T21E12.4 | *dhc-1* | C-terminal gfp | CTACCAACGAGGAGTTGCATTGG |
| ZK593.5 | *dnc-1* | F26L | GACAAACGCAATTCGCAGAAGG |
|  |  |  | CAATTCGCAGAAGGTGATTGGG |
|  |  |  | TCGCAGAAGGTGATTGGGTTGG |
| ZK593.5 | *dnc-1* | G33S | GACAAACGCAATTCGCAGAAGG |
|  |  |  | CAATTCGCAGAAGGTGATTGGG |
|  |  |  | TCGCAGAAGGTGATTGGGTTGG |
| ZK593.5 | *dnc-1* | G45R | GACAAACGCAATTCGCAGAAGG |
|  |  |  | CAATTCGCAGAAGGTGATTGGG |
|  |  |  | TCGCAGAAGGTGATTGGGTTGG |
| ZK593.5 | *dnc-1* | exon 4 & 5 fusion | AAAAAGAACTCCGAGTCAACGG |
|  |  |  | GCATAGATTACCGTTGACTCGG |
|  |  |  | TCGTTGTTTCAGTCGAGAATGG |
| ZK593.5 | *dnc-1* | exon 5 & 6 fusion | TGAGCATTATACAAACCCTAGG |
|  |  |  | GCGGCTGAAAATATTACATTGG |
| ZK593.5 | *dnc-1* | Δexon 4-5 | AGACCGCTTTTTCTATAAAAGG |
|  |  |  | TGAGCATTATACAAACCCTAGG |
| ZK593.5 | *dnc-1* | Δexon 4 | AGACCGCTTTTTCTATAAAAGG |
|  |  |  | AAAAAGAACTCCGAGTCAACGG |
|  |  |  | GCATAGATTACCGTTGACTCGG |
| ZK593.5 | *dnc-1* | exon 3 & 4 fusion | AAGCTGGCAGATCCCAATGAGG |
|  |  |  | AGACCGCTTTTTCTATAAAAGG |
| ZK593.5 | *dnc-1* | Δexon 5 | TCGTTGTTTCAGTCGAGAATGG |
|  |  |  | TGAGCATTATACAAACCCTAGG |
| ZK593.5 | *dnc-1* | Premature stop codon  (null mutant) | TCCTATTTCGAACGACATCTGG |
|  |  |  | GTCGTTCGAAATAGGAACTCGG |
|  |  |  | TCGTTCGAAATAGGAACTCGGG |
| ZK593.5 | *dnc-1* | C-terminal 3xflag | GAAGAAATCAGTGCCAAGTGGG |
|  |  |  | TGCCAAGTGGGGAATCGTGTGG |
|  |  |  | TACCACACGATTCCCCACTTGG |
