# Supplementary material for: Dynactin binding to tyrosinated microtubules promotes centrosome centration in C. elegans by enhancing dynein-mediated organelle transport
Source: PLoS Genet. 2017 Jul 31;13(7):e1006941. doi: 10.1371/journal.pgen.1006941 (PMC5552355; doi:10.1371/journal.pgen.1006941)
Supplement: S3 Table — (DOCX) [file pgen.1006941.s020.docx]

**S3 Table. Oligos for double-stranded RNA production.**

| Gene ID | Gene Name | Oligonucleotide 1  (T3 promoter) | Oligonucleotide 2  (T7 promoter) | Template |
| --- | --- | --- | --- | --- |
| C17H12.1 | *dyci-1* | AATTAACCCTCACTAAAGGGGCAACTTTCGACTTGTCA | TAATACGACTCACTATAGGCTCTTGCAAAGTAATCAG | cDNA |
| C38C10.4 | *gpr-2* | AATTAACCCTCACTAAAGGTCTGGCAGCAGACAGTTCAG | TAATACGACTCACTATAGGAGCATGTGATTCCACACGTC | gDNA |
| C50F4.11 | *mdf-1* | AATTAACCCTCACTAAAGGAGCATCCTCAAGTCGTTCGT | TAATACGACTCACTATAGGAAGCGAAGTTGGCTGAAAAA | gDNA |
| F22B7.13 | *gpr-1* | AATTAACCCTCACTAAAGGTCTGGCAGCAGACAGTTCAG | TAATACGACTCACTATAGGAGCATGTGATTCCACACGTC | gDNA |
| T03F6.5 | *lis-1* | AATTAACCCTCACTAAAGGTCGGAGAGGCAAAAAGAAGA | TAATACGACTCACTATAGGCTCGAACCCAATTTTCGTGT | cDNA |
| VW02B12L.3 | *ebp-2* | AATTAACCCTCACTAAAGGTCACCAAAGTGGAAGAGATGG | TAATACGACTCACTATAGGAACTGTCGGTGGAACTGATG | gDNA |
| Y59A8B.7 | *ebp-1* | AATTAACCCTCACTAAAGGCAAGCACGTAATGGAGAAGGT | TAATACGACTCACTATAGGGGTGGTGCGAATCCTTCTT | cDNA |
| Y59A8B.9 | *ebp-3* | AATTAACCCTCACTAAAGGCAAGCACGTAATGGAGAAGGT | TAATACGACTCACTATAGGGGTGGTGCGAATCCTTCTT | cDNA |
| ZK593.5 | *dnc-1* | AATTAACCCTCACTAAAGGTGCCTCTTGTGCAGCTTG | TAATACGACTCACTATAGGCCGCTGGAGAGGTATACAACA | cDNA |
